# Differential tissue growth and cell adhesion alone drive early tooth morphogenesis: An *ex vivo* and *in silico* study

**DOI:** 10.1371/journal.pcbi.1005981

**Published:** 2018-02-26

**Authors:** Miquel Marin-Riera, Jacqueline Moustakas-Verho, Yoland Savriama, Jukka Jernvall, Isaac Salazar-Ciudad

**Affiliations:** 1 Department of Genetics and Microbiology, Universitat Autònoma de Barcelona, Barcelona, Spain; 2 Centre of Excellence in Experimental and Computational Developmental Biology, Institute of Biotechnology, University of Helsinki, Helsinki, Finland; 3 Centre for Genomic Regulation, Barcelona Institute of Science and Technology, Barcelona, Spain; University of California Irvine, UNITED STATES

## Abstract

From gastrulation to late organogenesis animal development involves many genetic and bio-mechanical interactions between epithelial and mesenchymal tissues. Ectodermal organs, such as hairs, feathers and teeth are well studied examples of organs whose development is based on epithelial-mesenchymal interactions. These develop from a similar primordium through an epithelial folding and its interaction with the mesenchyme. Despite extensive knowledge on the molecular pathways involved, little is known about the role of bio-mechanical processes in the morphogenesis of these organs. We propose a simple computational model for the biomechanics of one such organ, the tooth, and contrast its predictions against cell-tracking experiments, mechanical relaxation experiments and the observed tooth shape changes over developmental time. We found that two biomechanical processes, differential tissue growth and differential cell adhesion, were enough, in the model, for the development of the 3D morphology of the early tooth germ. This was largely determined by the length and direction of growth of the cervical loops, lateral folds of the enamel epithelium. The formation of these cervical loops was found to require accelerated epithelial growth relative to other tissues and their direction of growth depended on specific differential adhesion between the three tooth tissues. These two processes and geometrical constraints in early tooth bud also explained the shape asymmetry between the lateral cervical loops and those forming in the anterior and posterior of the tooth. By performing mechanical perturbations *ex vivo* and *in silico* we inferred the distribution and direction of tensile stresses in the mesenchyme that restricted cervical loop lateral growth and forced them to grow downwards. Overall our study suggests detailed quantitative explanations for how bio-mechanical processes lead to specific morphological 3D changes over developmental time.

## Author summary

The genes and signalling pathways involved in ectodermal organ development (teeth, hair, mammary glands, etc.) are relatively well studied. However, the bio-mechanical processes by which these organs grow and change shape from an early primordium (an epithelial bud similar among different organs) is far less understood, especially in mammalian development. This study combines simple experiments and a multi-scale, cell-based computational model to understand these processes for one ectodermal organ: the tooth. Our model implements the different mechanical properties of the different cell types involved in morphogenesis (i.e. epithelial and mesencymal) and their interactions. By exploring model behaviour and contrasting it with experimental data on tissue growth rates and mechanics we found that, in spite of their relative complexity, the shape changes occurring in early tooth development, the overall sharpness of the tooth and its variation can largely be explained by the two most simple bio-mechanical processes: differential growth and differential adhesion between tooth tissues. These results suggest that simple mechanical interactions between cells and tissues may underlie the complex tissue deformations observed in the morphogenesis of several ectodermal organs.

## Introduction

From gastrulation to late organogenesis, animal development involves many examples of reciprocal genetic and biomechanical interactions between epithelial and mesenchymal tissues [1]. Ectodermal organs, such as hairs, feathers, teeth and mammary glands are easily accessible examples of organs whose development is based on epithelial-mesenchymal interactions. Despite the diversity in their mature form and function, ectodermal organs share several common features during the early steps of development [2–4]. Organogenesis begins with the appearance of local epithelial thickenings, placodes, followed by condensation of the underlying mesenchymal cells. Later, the placode develops into a bud that grows into or out of the mesenchyme ensued by further growth and morphogenesis specific to each organ [2]. The genetic regulation of this latter process is relatively well understood [2, 3], whereas the contributing cellular and bio-mechanical mechanisms have remained largely unexplored. In this study we explore this latter question for tooth development as an example ectodermal organ.

**Fig 1:**
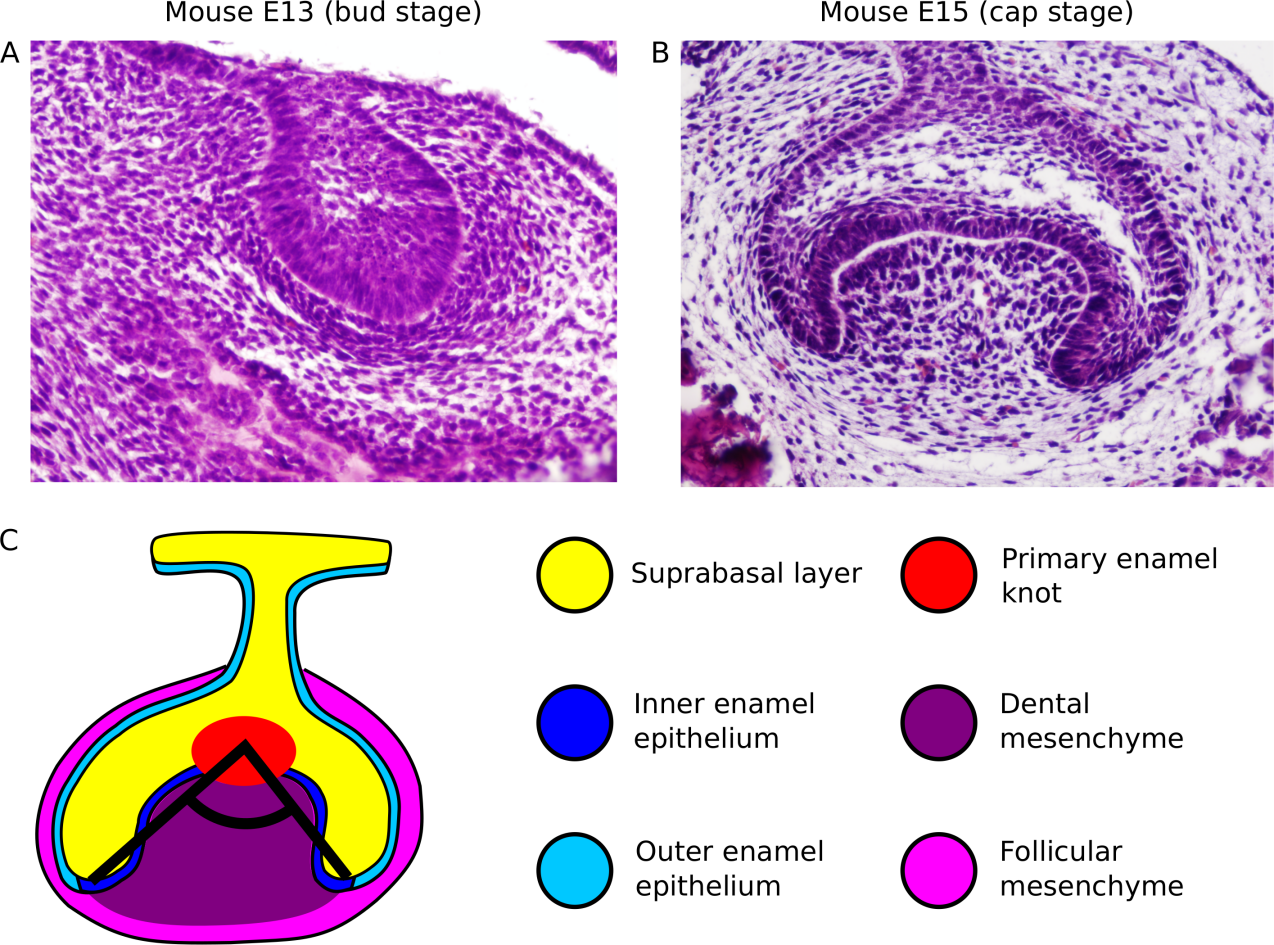
Stages of tooth development considered for this study. A, Bud stage of the mouse first molar (E13), shown as a frontal section (HE staining). B, Cap stage of the mouse first molar (E15.5), shown as a frontal section (HE staining). C, Diagram depicting the growth angle of the cervical loops (black lines) in a cap stage tooth germ (frontal section). Color legend indicates different tissue types.

Teeth develop through a complex process which combines cell signalling, extensive cell movements and tissue deformation [5–8]. At the late bud stage (Fig 1A), a signalling centre appears in the epithelium, the primary enamel knot, and a mesenchymal condensate forms beneath the epithelial bud. This is followed by the emergence of two epithelial folds, the buccal and lingual cervical loops, on the respective sides of the tooth germ. These initially grow laterally but progressively change their orientation, or angle of growth, downwards (cap stage, Fig 1B). The angle of growth of the cervical loops is defined here as the angle between the tips of the cervical loops and the primary enamel knot in a frontal plane (Fig 1C). The cervical loops largely delineate the boundaries of the tooth germ. The epithelium between the cervical loops is called the inner enamel epithelium and its growth and folding largely determine the overall shape of the tooth crown. The rest of the epithelial sheet, called the outer enamel epithelium, will not form part of the tooth crown. Similarly, the mesenchyme enclosed by the cervical loops, called the dental mesenchyme, will become part of the tooth crown, whereas the mesenchyme in contact with the outer enamel epithelium, called the follicular mesenchyme, will not. The tissue located on the apical side of the enamel epithelium is called the suprabasal layer and it is already present at the earliest stages of tooth development (Fig 1A). At the late cap stage, secondary enamel knots form in the inner enamel epithelium and a cusp will arise under each one of them [9]. The tooth germ progressively elongates in the anterior-posterior axis and cervical loops form in the anterior and the posterior of the tooth germ as a continuation of the buccal and lingual cervical loops. The angle of growth of the cervical loops has a general effect on the sharpness of the tooth, with smaller angles leading to sharper teeth [10], and in some species on the positioning of secondary enamel knots [11]. At later stages, enamel secreting ameloblasts and dentin secreting odontoblasts differentiate at the interface between the inner enamel epithelium and mesenchyme to form the mineralized tooth.

In this study we build a new mathematical model of early tooth development. Our aim is to understand how the tooth germ changes in shape from the bud to the cap stage. This implies understanding how the cervical loops form, what determines the orientation of their growth in the different parts of the tooth germ and how that affects the shape of the tooth germ. Theoretical studies in development and tissue mechanics predict that epithelial folding can be the result of differential growth between adjacent tissues [12–14]. For example, differential growth between the epithelium and the suprabasal layer modelled in 2D has been used to produce buckling reminiscent of the bud to cap stage transition [14]. Although not considering biomechanical properties such as differential adhesion, the previous studies underscore the requirement of differential growth in the progression of epithelial development beyond the bud stage.

Here we hypothesize that the growth and orientation of the cervical loops depends on both the differential growth and differential adhesion between the epithelium, the suprabasal layer, and the mesenchyme. Variation in differential growth and adhesion should then lead to variation in the orientation of the cervical loops. We implement this hypothesis in a new 3D cell-based model of tooth morphogenesis using the recent EmbryoMaker modelling framework of animal development [15] (see S1 Appendix). EmbryoMaker is essentially a modelling tool that uses a mathematical implementation of the basic cellular and molecular processes known in animal development. We use this modelling framework to build a tooth specific model by specifying to EmbryoMaker the distribution of cell types at the bud stage, gene expression dynamics and signalling, and the cell behaviours involved in early tooth development. This study, however, does not restrict itself to study the wild-type mouse molar. The aim is also to understand how the length and orientation of the cervical loops in 3D changes with differential growth and adhesion.

There are several previous mathematical models of tooth development [16, 17]. These models implement only the inner enamel epithelium and cannot be used to model the cervical loops and the dynamics that define the boundary between inner and outer enamel epithelium. Furthermore, these earlier models do not explicitly consider the three main tooth germ tissues, epithelium, mesenchyme, and suprabasal layer and, thus, cannot study, in an integrated way, how their differential adhesion and growth affect early tooth development. In addition, EmbryoMaker implements much more refined cell and tissue biomechanics than previous models, thereby allowing us to decompose the different roles of tissue growth and adhesion dynamics.

## Results

We used the EmbryoMaker modelling framework [15] to implement our tooth-specific model. Cells are represented as single or sets of multiple particles (herein called nodes) with specific physical properties. For the purpose of this model, mesenchymal and suprabasal cells are represented as single spherical nodes, whereas epithelial cells are represented as a cylindrical body consisting of two nodes (one basal and one apical bound by an elastic link) (S1 Fig). Each node interacts with its immediate neighbouring nodes by exerting repulsive and attractive forces depending on whether the two nodes are closer or farther away from each other, respectively. An equilibrium distance determines the separatation between the repulsive and the attractive regime, and a cut-off distance determines the point at which cells lose contact (S1A and S1B Fig). Apart from the previously mentioned, epithelial cells exert additional force components in order to reflect their specific mechanical properties and their organisation in epithelial sheets (S1C–S1F Fig) (see Methods section and [15]). Cell-cell adhesion can be modulated by changing the strength of the attractive force between two nodes and tissue growth is modulated by local cell division events, which consist on duplicating an existing cell by adding new nodes. For more details on the basics of EmbryoMaker see the Methods section and [15].

Below we explain the specific implementation of the tooth model using EmbryoMaker. It consists simply of specifying to EmbryoMaker what initial condition to use, what is the adhesion between and within the three tissues, what are the growth rates of each tissue, and how the latter are regulated in space by growth factors secreted from the primary enamel knot. These are the only things specified to the model; how tooth morphology changes over developmental time is a result of the model dynamics. The tooth model covers the time span between the bud and cap stages (E13-E15, Fig 1A and 1B).

**Fig 2:**
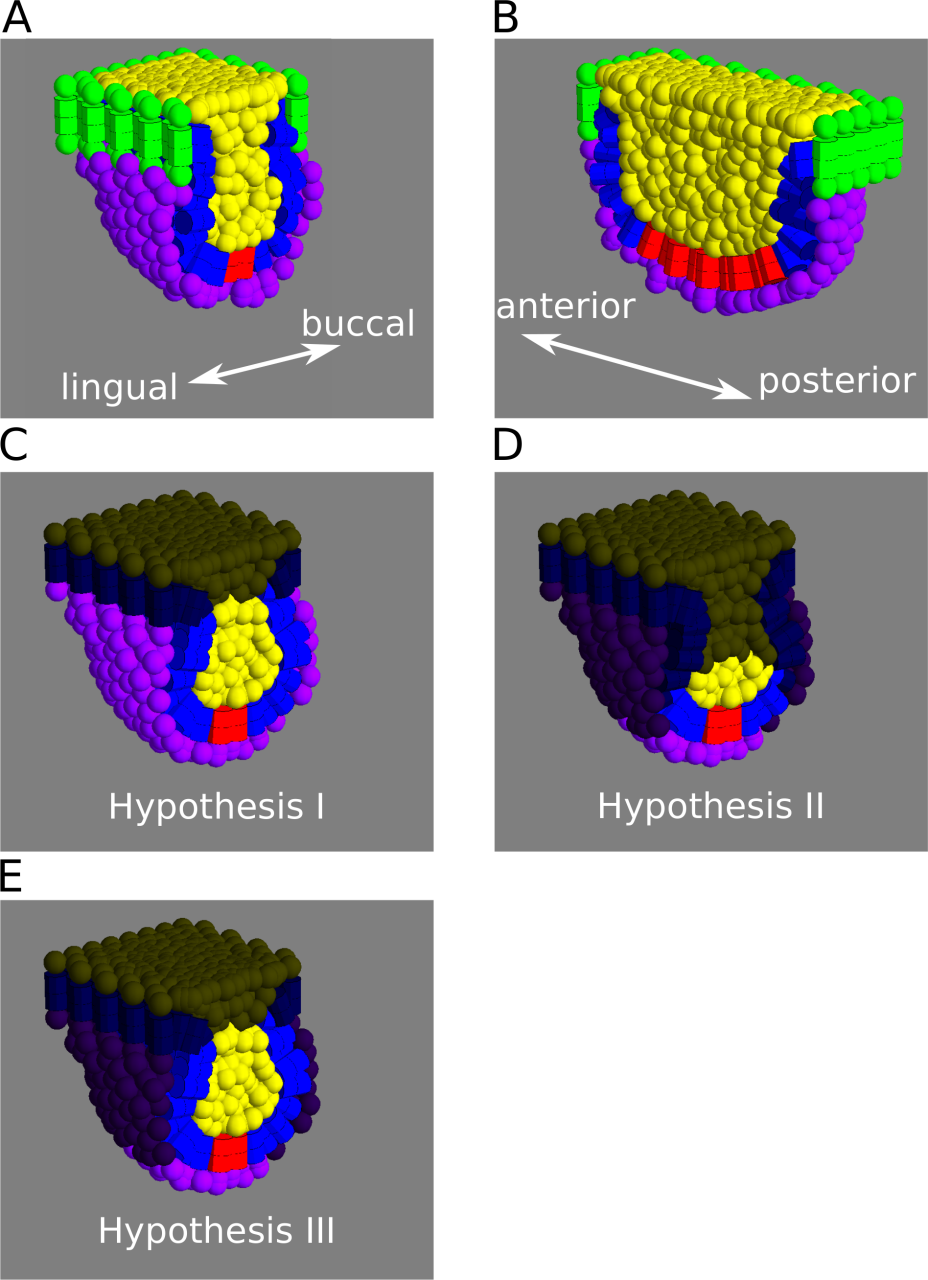
Tooth model initial conditions. A, B, Depiction of the model initial conditions as frontal (A), and sagittal (B) sections. The cells lining the border of the epithelium (cells in green) and the uppermost layer of suprabasal cells are fixed in space, representing the physical barrier imposed by the oral epithelium surrounding the base of the tooth germ. The space under the mesenchymal layer however, is empty, allowing the tooth germ to grow in depth, bucco-lingually and antero-posteriorly. C-E, The three hypotheses raised as different distributions of cell proliferation in the different tissues, frontal sections shown. Non proliferative cells have been darkened. Blue: enamel epithelium. Yellow: Suprabasal layer. Purple: mesenchyme. Red: enamel knot.

We set up the initial condition to mimic the spatial arrangement of the three different tissues at the bud stage (Fig 2A and 2B). We specify the primary enamel knot as a group of cells located at the mid line of the inner enamel epithelium. This group of cells keep their identity throughout the simulations and do not divide (as observed experimentally, [9]).

Even though there is no direct biological evidence of differential adhesion between tooth epithelium, suprabasal layer and mesenchyme, it has been shown that different types of adhesion molecule are expressed in each of these tissues at different stages of tooth development [18, 19]. Thus, we assume that cells have tissue-specific (i.e. epithelial, suprabasal or mesenchymal) cell-cell adhesion properties (see S1 Appendix for more details). Differential cell adhesion can be modulated in different runs of the model by changing the values of a symmetric 3x3 *B* matrix. Each element in this matrix specifies the adhesion strength between pairs of cell types. For example, *b*_*EE*_ is the homotypic adhesion between epithelial cells, while *b*_*EM*_ is the heterotypic adhesion between epithelial and mesenchymal cells (see Materials and Methods, Eq 3).

Imaging of tooth morphogenesis in Fucci transgenic mice [20], where cells express two different fluorescent proteins depending on whether they are in a proliferative state (stage G2/S/M of cell cycle) or not (G0/G1), indicates that cells proliferate everywhere in the epithelium, suprabasal layer and mesenchyme, except for the primary enamel knot in the epithelium [6]. From Fucci labelling, however, it is quite difficult to know how rapidly cells divide. It could be, for example, that cells close to the enamel knot proliferate at a faster rate than cells far away from it because they receive higher concentrations of the growth factors known to be secreted from the knot [21].

We proposed three different hypotheses on how cell proliferation is distributed across tissues. In hypothesis I (Fig 2C), we assume that cells within each tissue divide at the same rate and that rate is different in different tissues, so differential growth between tissues can be modulated in the model by changing these rates between simulations. These are *s*_*epi*_ for epithelium, *s*_*sup*_ for suprabasal cells and *s*_*mes*_ for mesenchyme (see S1 Appendix for details). These parameters are expressed as the inverse of the cell cycle length (that is in hours^-1^). In hypothesis II (Fig 2D), cells in the primary knots secrete an extracellular diffusible signal that induces proliferation. Cells that receive the signal above a certain threshold concentration proliferate at a certain rate (*s*_*epi*_, *s*_*sup*_ or *s*_*mes*_) whereas cells that receive it under the threshold do not proliferate at all (see S1 Appendix). This means, that at any time, only cells that are close enough to the signalling centre will proliferate. In hypothesis III (Fig 2E), we use ubiquitous cell proliferation for the epithelium and suprabasal layer (as in hypothesis I) and localised proliferation for the mesenchyme (as in hypothesis II).

In order to assess the ability of the model to generate tooth germ-like morphologies, we performed a series of parameter screenings for sub-sets of model parameters. For each screening, a representative list of values was chosen for each of the selected parameters (reasonable boundaries for parameter values were set by manual search) and the model was run for all the permutations of these parameter values. Non-selected parameters were kept at a constant, intermediate value. We performed a parameter screening for the tissue growth parameters (see below in the Results section), another for the cell-cell adhesion parameters (see below in the Results section) and two more focusing on other tissue-specific parameters (S2 Fig). More specifically we varied the cell imcompressibility parameter (*p*^*REC*^, see Methods) in a tissue-specific fashion (i.e. for each cell type independently) (S2A–S2C Fig) and, in a different screening, the two epithelial bending parameters (*p*^*EST*^ and *p*^*ERP*^, see Methods) (S2D Fig). The result of the two latter searches indicated that those parameters had a small effect on the generation of shape in the tooth germ, so we focused our analysis on the tissue-specific growth and adhesion parameter screenings.

### The tooth cap arises through differential growth of the epithelium, suprabasal layer and mesenchyme

**Fig 3:**
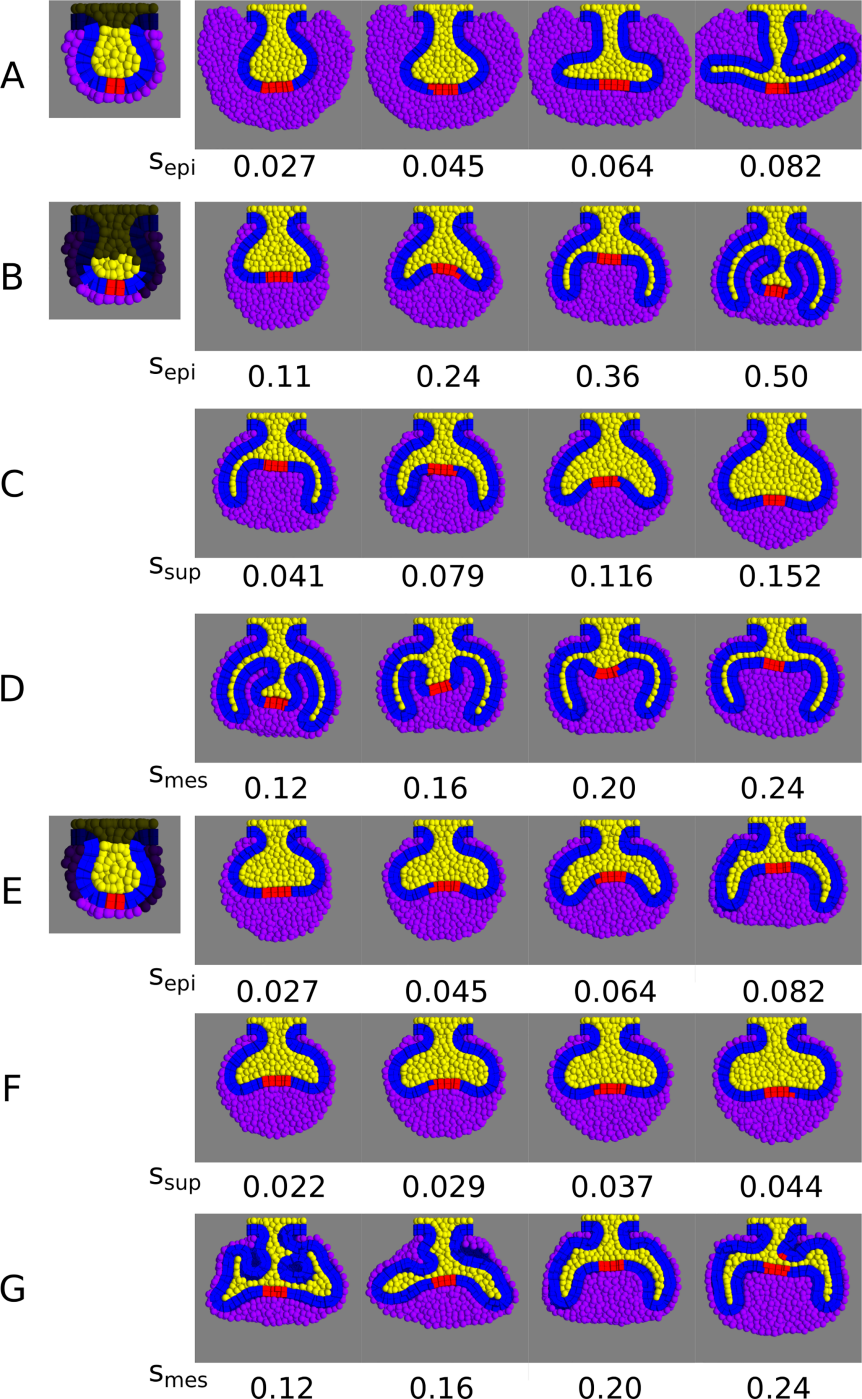
Parameter screening of the tissue specific proliferation rates (s_epi_, s_sup_ and s_mes_). Variation in tooth germ morphology with different combinations of either *s*_*epi*_, *s*_*sup*_ *or s*_*mes*_, keeping the other two constant, under the different hypotheses (frontal sections depicted). A, B, E, variation in *s*_*epi*_ for hypothesis I, II and III respectively (initial conditions depicted on the left). A, *s*_*sup*_ = 0.022, *s*_*mes*_ = 0.10. B, *s*_*sup*_ = 0.041, *s*_*mes*_ = 0.20, E, *s*_*sup*_ = 0.029, *s*_*mes*_ = 0.20. In all cases, cervical loops form when *s*_*epi*_ is relatively high and *s*_*sup*_ is relatively low, but only in hypotheses II and III these are oriented downwards as in tooth development. C, F, Variation in *s*_*sup*_ for hypotheses II and III respectively. C, *s*_*epi*_ = 0.36, *s*_*mes*_ = 0.20. F, *s*_*epi*_ = 0.045, *s*_*mes*_ = 0.20. Increasing values of *s*_*sup*_ prevent the formation of cervical loops. D, G, Variation in tooth morphology for different values of *s*_*mes*_ under hypotheses II and III respectively. D, *s*_*epi*_ = 0.50, *s*_*sup*_ = 0.041. G, *s*_*epi*_ = 0.082, *s*_*sup*_ = 0.029. A relatively high mesenchymal proliferation is necessary for the proper formation of the cervical loops. Colours as in Fig 2. *s*_*epi*_, *s*_*sup*_ and *s*_*mes*_ values are expressed in *h*^*-1*^.

In order to assess which hypotheses were capable of reaching cap morphology, we performed a parameter screening on the tissue-specific growth parameters. We ran the tooth-specific model under different combinations of tissue growth rates (*s*_*epi*_, *s*_*sup*_ and *s*_*mes*_ parameters) and under the three different hypotheses (Fig 3, S3 Fig). For all hypotheses, we found that the cervical loops appeared at the buccal and lingual sides of the tooth germ when the proliferation rate in the epithelium was high relative to the suprabasal and mesenchymal proliferation rates (Fig 3A, 3B and 3E). The length of these loops increased with the epithelial to suprabasal proliferation ratio (S3A–S3C Fig). High suprabasal growth rates relative to the epithelium growth rates led to enlarged buds lacking cervical loops (Fig 3C and 3F). However, only in hypotheses II and III (Fig 3B and 3E) did the cervical loops grow downwards similarly to the wild-type (small growth angle). In hypothesis I, the cervical loops grew with an angle close to 180° (Fig 3A), and the follicular mesenchyme was roughly as thick as the dental mesenchyme, unlike wild-type tooth germs (Fig 1B). Thus, we rejected hypothesis I and concluded that the formation of the cervical loops requires the proliferation rate of the follicular mesenchyme to be lower than that of the dental mesenchyme. In hypothesis II, low mesenchymal growth (*s*_*mes*_<0.20) led to an abnormal shape of the dental mesenchyme, with additional epithelial folds between the cervical loops (Fig 3D). In hypothesis III, the height of the dental mesenchyme (the difference in height between the tip of the cervical loops and the base of the signalling centre) increased with the proliferation rate of the mesenchyme (Fig 3G). Thus, in hypotheses II and III, a sufficiently high proliferation of the dental mesenchyme is necessary for normal cap morphology to arise.

**Fig 4:**
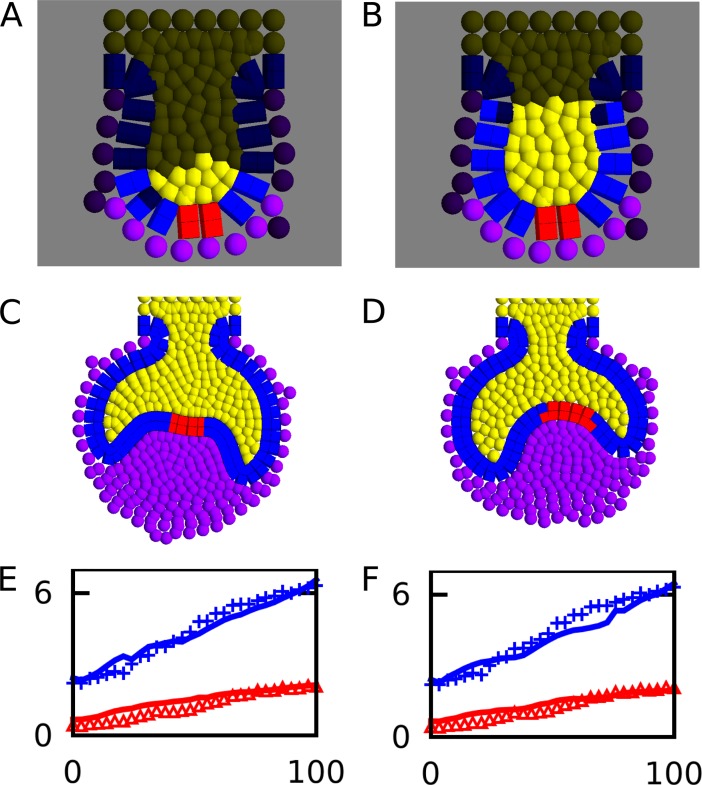
Comparison of growth rates with experimental data. A, B, Initial conditions for the 2D tooth development model for hypothesis II and III respectively. Distribution of proliferating cells for each hypothesis is indicated with the same colours as in Fig 2. Examples of best fitting tooth germ morphologies for hypothesis II (*s*_*epi*_ = 0.36, *s*_*sup*_ = 0.11, *s*_*mes*_ = 0.20) and III (*s*_*epi*_ = 0.05, *s*_*sup*_ = 0.03, *s*_*mes*_ = 0.25) respectively. E, F, Epithelium perimeter (blue) and surface area (red) measured over developmental time for the teeth shown in C and D respectively, compared to the empirical growth curves (blue crosses and red triangles) from tooth sections (6). X axis shows time in percentual units, Y axis represents size (see S1 Appendix for details).

Next we wanted to test whether the model was able to create a realistic cap morphology with tissue growth rates estimated from experimental observations. We measured the length of the dental epithelium and the surface area of the suprabasal layer from the time-lapse sequence of a developing mouse molar recently published by Morita and collaborators [6] at different time points, thus creating a growth curve for both the epithelium and the suprabasal layer. The time-lapse in Morita *et al*. was performed on a molar thick frontal section, thus the data was essentially 2D. In order to better compare that data to our model, we created a 2D version of the tooth model (Fig 4A and 4B), based on the same principles as the original model, and performed a large parameter screening of tissue growth and adhesion parameters. For each simulation we calculated the growth curves of the epithelium and the suprabasal layer, and then compared them to the empirical ones by calculating the standard error between the theoretical and empirical curves (S4 Fig, see S1 Appendix). We performed a sensitivity analysis of the standard error measurement against each of the parameters chosen for the screening (S5 Fig). In both hypothesis II and III, the model showed the largest sensitivity on the epithelial growth parameter (*s*_*epi*_) (S5A and S5E Fig), whereas it showed the lowest sensitivity to the mesenchymal growth parameter (*s*_*mes*_) and the five adhesion parameters (S5C, S5D, S5G and S5H Fig). In both hypotheses II and III, a relatively large number of model runs (approximately 500 in each case) had a good fit (standard error < 10) with the empirical growth curves. Visual inspection of the morphology in these better fitting subsets showed that the great majority of model runs achieved a proper cap morphology in the 2D model (e.g. Fig 4C–4F).

**Fig 5:**
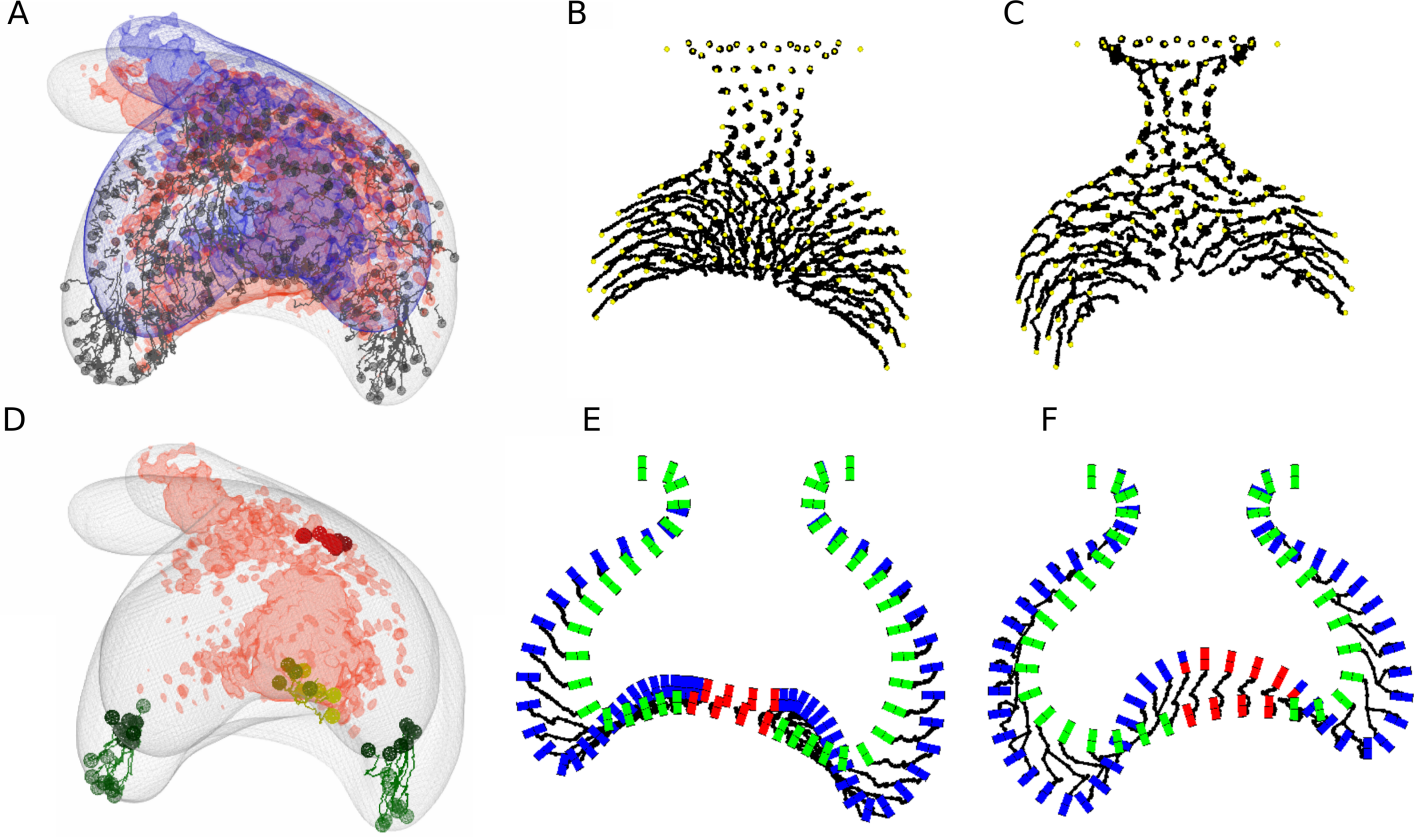
Comparison of cell movements with experimental data. A, Experimental cell trajectories as shown in (6) (with permission). B, C, *in silico* trajectories (black lines) of suprabasal cells for hypothesis II and III respectively (same simulations as in Fig 4). D, empirical cell trajectories from Morita et al., highlighting a group of epithelial cells at the tip of each cervical loop. E, F, *in silico* trajectories of epithelial cells only for the morphologies shown in B and C respectively. Epithelial cells are shown before (green cylinders) and after (blue cylinder) the tracking interval and cell trajectories are shown as black lines. In hypothesis II (E), cells that were previously at the tip of the cervical loops move away from it and are replaced by cells coming from the centre by the end of the tracking period. In contrast, in hypothesis III (F) epithelial cells that start at the tip of the loops stay there after the tracking period, which is more consistent with the experimental data (D).

Even if the model predicted correctly the overall morphology of the tooth germ, it could be that this was accomplished by different patterns of cell movement compared to the real tooth. We proceeded to compare cell movement in the 2D model (see S1 Appendix) with the empirical cell trajectories during tooth development [6]. In their study, Morita et al. recorded multiple cell trajectories on the epithelium and suprabasal layer during their time-lapse (Fig 5A and 5D, see also [6]). In our model, suprabasal cells showed a consistent movement towards the tips of the cervical loops in both hypotheses II and III (Fig 5B and 5C). We then decided to focus on the behaviour of epithelial cells. In Morita’s experiments, they tracked a small group of epithelial cells at the tip of both cervical loops, and they observed that by the end of the tracking period they still remained at the tip, even though the tissue had grown significantly (Fig 5D). When looking at the movement of epithelial cells in our simulations, differences were observed between hypothesis II and III. In hypothesis II, cells that were at the tip of the cervical loops at the start of the tracking period had moved outwards by the end of the tracking, and cells that were medially located at the start moved to the tip of the cervical loops (Fig 5E). Lateral movement of epithelial cells in II was caused by the fact that cells only proliferated close to the enamel knot and slid sideways as the epithelium grew (Fig 5E). In hypothesis III, cells that started at the tip of the cervical loops remained in them by the end of the tracking period (Fig 5F). Thus, it seems unlikely that epithelial cells proliferate only near to the enamel knot, since the cell movements observed *ex vivo* should be different than the ones presented by Morita and collaborators. Thus, we decided to reject hypothesis II and, for the rest of the study we only show the computational analyses corresponding to hypothesis III. Note, however, that the analyses described in the following sections were also performed for hypothesis II and led to the same conclusions than III, but we chose to exclude them for the sake of simplicity.

### Differential cell adhesion regulates length and orientation of the cervical loops

**Fig 6:**
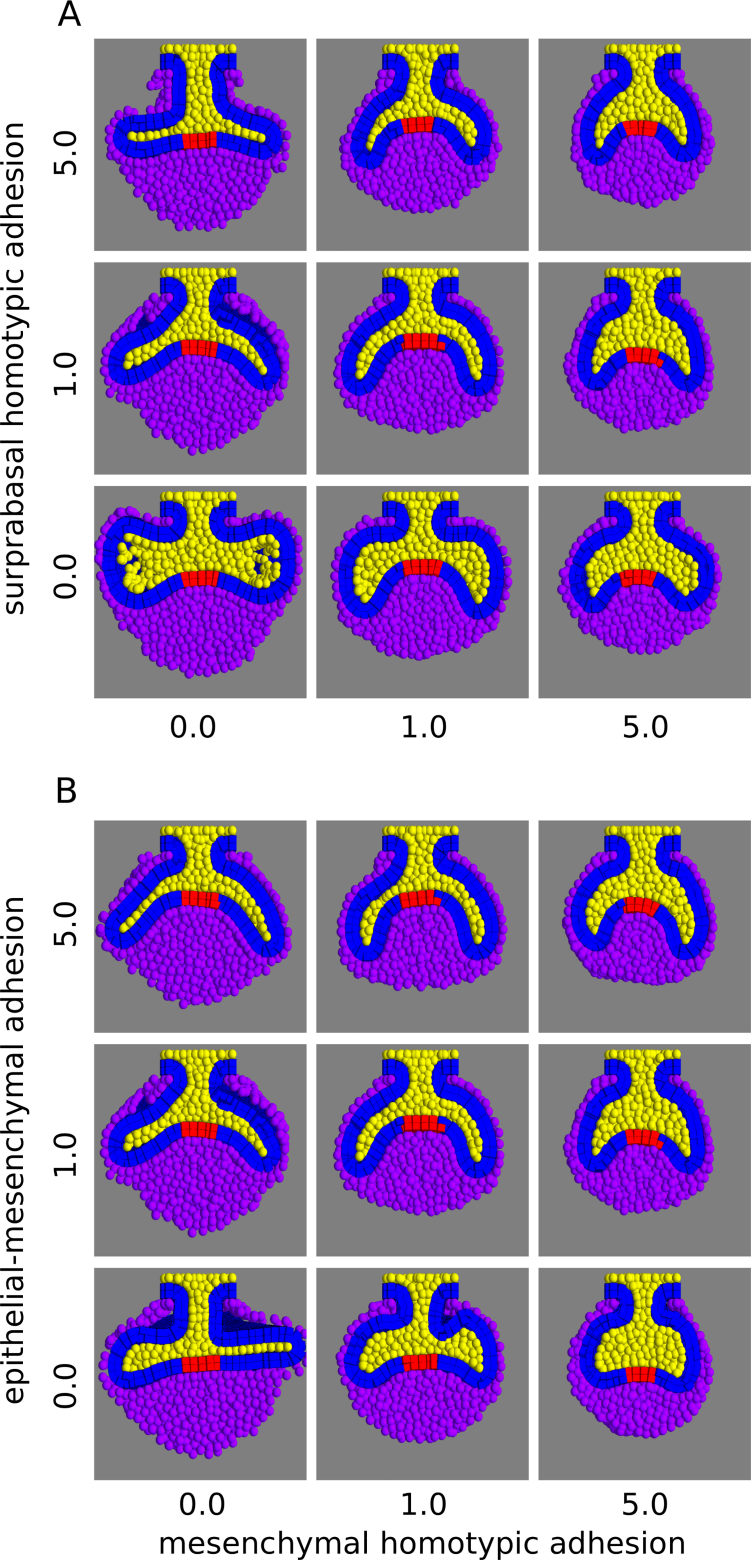
Parameter screening of differential adhesion parameters. A, Tooth germ morphologies are shown with different combinations of mesenchymal homotypic adhesion and suprabasal homotypic adhesion values, while the other parameters are set to 1.0. High values of mesenchymal homotypic adhesion result in low growth angles whereas high values of suprabasal homotypic adhesion results in high growth angles. B, Morphologies are shown with different combinations of mesenchymal homotypic adhesion and epithelial-mesenchymal adhesion values (other adhesion parameters set to 1.0). High values of epithelial-mesenchymal adhesion result in low growth angles. Frontal sections shown. Colours as in Fig 3. All the simulations in this screening used the following growth parameters: *s*_*epi*_ = 0.063, *s*_*sup*_ = 0.021, *s*_*mes*_ = 0.182.

We performed a parameter screening in the 3D model for the five adhesion parameters: epithelium homotypic (*b*_*EE*_), epithelium-suprabasal (*b*_*ES*_), epithelium-mesenchyme (*b*_*EM*_), suprabasal homotypic (*b*_*SS*_), and mesenchyme homotypic adhesion (*b*_*MM*_). The resulting morphologies displayed differences in the length and orientation of the cervical loops (Fig 6, S6 Fig). By looking at the frontal sections, we observed that high mesenchyme homotypic adhesion led to short cervical loops oriented downwards (small growth angle), whereas low values led to long cervical loops oriented bucco-lingually (large growth angle) (Fig 6A). The latter effect was more marked when the suprabasal homotypic adhesion was high (Fig 6A, top row). The cervical loops were also oriented downwards when the epithelium-mesenchyme adhesion was high, especially when the mesenchyme homotypic adhesion was also high (Fig 6B, top row). This seemed to occur because the epithelium deformed to maximize its contact area with the dental mesenchyme, even when the mesenchymal homotypic adhesion was null (e.g. Fig 6B left column). In contrast, with null epithelial-mesenchymal adhesion, the cervical loops failed to surround the dental mesenchyme, and the growth angle was high (Fig 6B bottom row).

**Fig 7:**
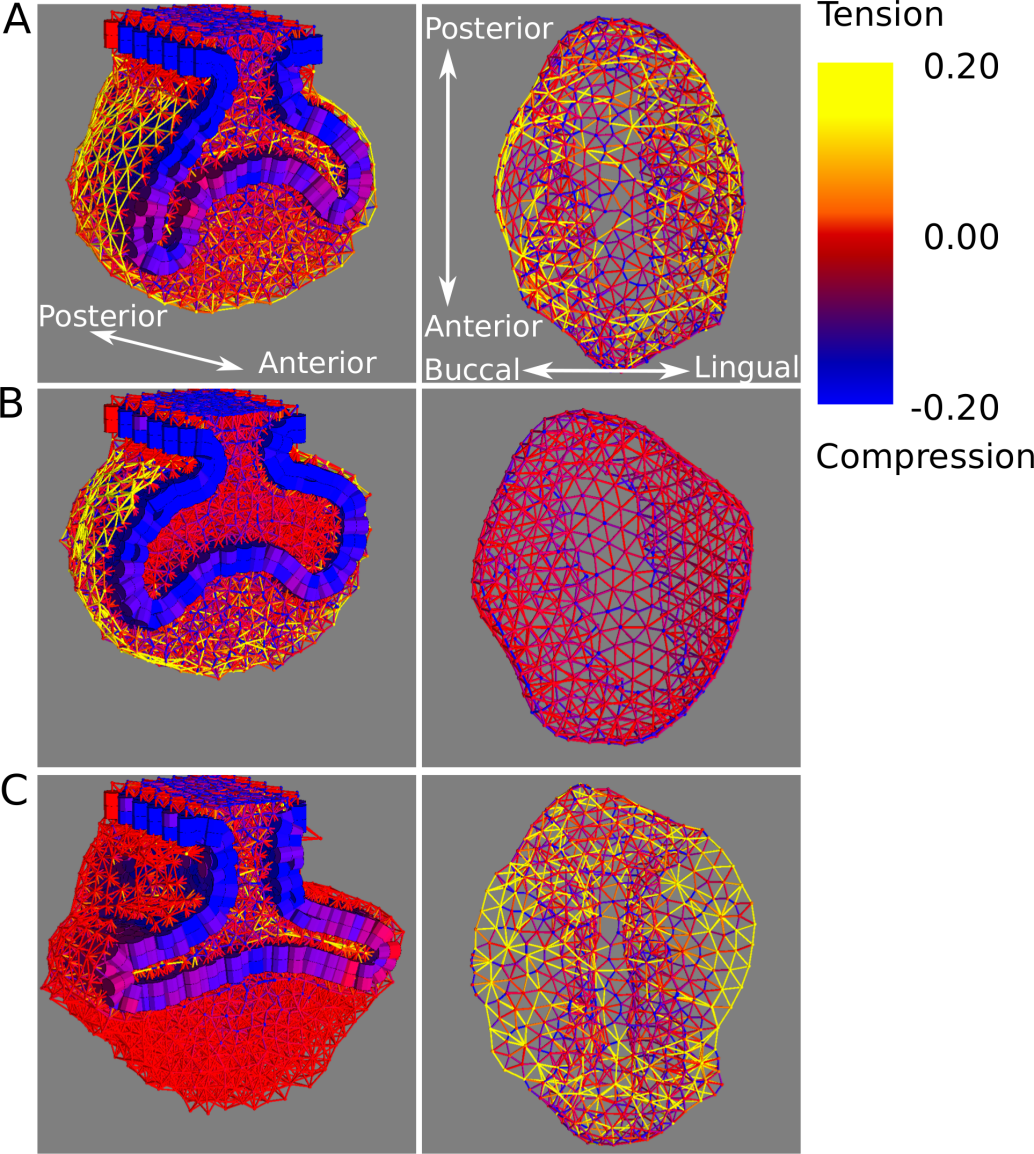
Mechanical forces acting on the different tooth tissues in the model. Spatial distribution of forces are shown for three different tooth morphologies at cap stage simulated with different adhesion parameters: *b*_*ss*_ = 5.0, *b*_*mm*_ = 1.0 (A), *b*_*ss*_ = 0.0, *b*_*mm*_ = 5.0 (B), *b*_*ss*_ = 5.0, *b*_*mm*_ = 0 (C), while the other adhesion parameters are equal to 1.0. Intensity and directionality of forces in the mesenchyme and suprabasal layer are depicted with rods connecting each pair of neighbouring cells. Rod colour indicates sign (i.e. negative = compression; positive = tension) and intensity of force. Rods are not displayed in epithelial cells, instead cylinder colour corresponds to the average of all the forces acting on it. For each simulation, frontal sections are shown from an oblique view (left column) and also the suprabasal tissue alone seen from below (right column, note that we only show the suprabasal cells in contact with the epithelium for a clearer picture of single rods). The growth parameters used in all three simulations are the same as for the simulations in Fig 6.

In order to understand how differential adhesion affects the shaping of the tooth germ, we plotted the spatial patterns of mechanical stress at the level of cell-cell contacts. We observed that there was a strong association between the orientation of the cervical loops and the spatial patterns of stress in the tooth germ. In simulations where high mesenchymal homotypic adhesion led to downwards oriented cervical loops, there was tension along the surface of the follicular mesenchyme surrounding the tooth germ (Fig 7A and 7B, left column). When that adhesion was null and the cervical loops grew in the bucco-lingual direction, there was no such tension in the mesenchyme (Fig 7C, left column). We also observed tension in the suprabasal layer in simulations where high suprabasal homotypic adhesion led to bucco-lingually oriented cervical loops (Fig 7A and 7C, right column). We did not observe high tension or compression at the interface between the epithelium and the suprabasal layer, nor between the epithelium and the mesenchyme when the epithelial-suprabasal and epithelial-mesenchymal heterotypic adhesions were high (S7 Fig).

### Differential growth of the cervical loops in the lingual and buccal side versus the anterior and posterior sides

**Fig 8:**
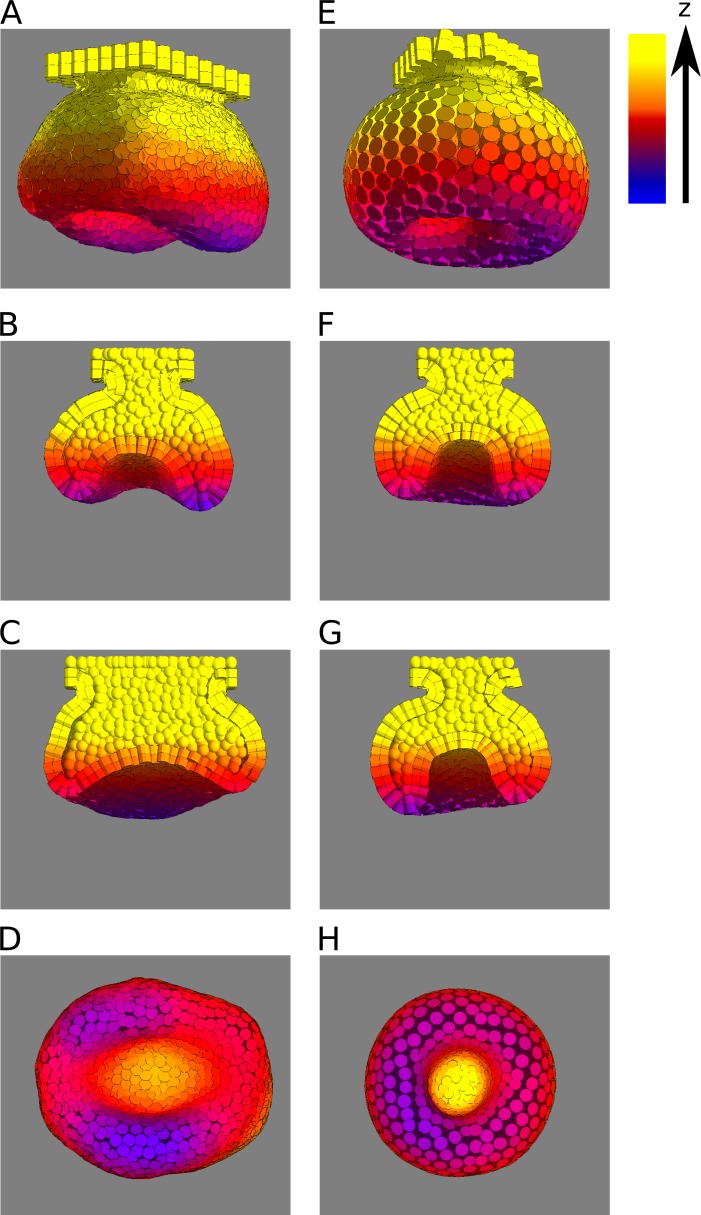
Anteror-posterior (AP) cervical loops tend to be shorter than bucco-lingual (BL) ones when the tooth germ at the initial conditions is longer in the AP axis than in the BL axis. A-D, an example simulation with initial conditions that are longer in the AP axis respect to the BL axis, seen from different angles: oblique (A), frontal section (B), sagittal section (C) and from below (D). Differences in the length of the cervical loops between the AP sides and BL sides can be observed. Growth and adhesion parameters used: *s*_*epi*_ = 0.063, *s*_*sup*_ = 0.021, *s*_*mes*_ = 0.182, *b*_*ee*_ = 1.0, *b*_*es*_ = 1.0, *b*_*em*_ = 5.0, *b*_*ss*_ = 1.0, *b*_*mm*_ = 5.0. E-H, an example simulation with initial conditions equally long in the AP and BL axes, seen from different angles: oblique (E), frontal section (F), sagittal section (G) and from below (H). It can be seen that in this case cervical loops have the same length on all sides. Growth parameters used: *s*_*epi*_ = 0.37, *s*_*sup*_ = 0.12, *s*_*mes*_ = 0.20, all adhesion parameters equal to 1. Only epithelium and suprabasal layer are shown. The colour code indicates the heigth (i.e. position in the Z axis).

Mouse molars are longer in the antero-posterior axis than in the bucco-lingual axis. This has been associated with cervical loops in the buccal and lingual sides forming earlier and growing more downwards than the cervical loops in the anterior and posterior sides [7, 9]. In the model, we observed that the cervical loops form in the same fashion in most cases (Fig 8A–8D), that is, also with an anterior-posterior versus bucco-lingual asymmetry. In other words, the overall shape of the epithelium in 3D is similar between mouse and model. For this to happen, however, the tooth germ in the initial condition needs to be at least slightly longer in anterior-posterior axis than wider in the bucco-lingual axis (as it is the case in mouse tooth germs from very early on, [7, 9]). If we choose initial conditions with radial symmetry, cervical loops grow equally in all directions, a mode of development that would produce a single fang or canine shaped tooth (Fig 8E–8H).

### Inferring patterns of mechanical stress ex vivo and in silico

Results from the model like those shown in Fig 6B suggest that strong adhesion between epithelium and mesenchyme results in downward oriented cervical loops. A prediction arising from the adhesion dynamics is that an experimental separation of the epithelium and the mesenchyme should lead to a fast increase of the cervical loop growth angle.

**Fig 9:**
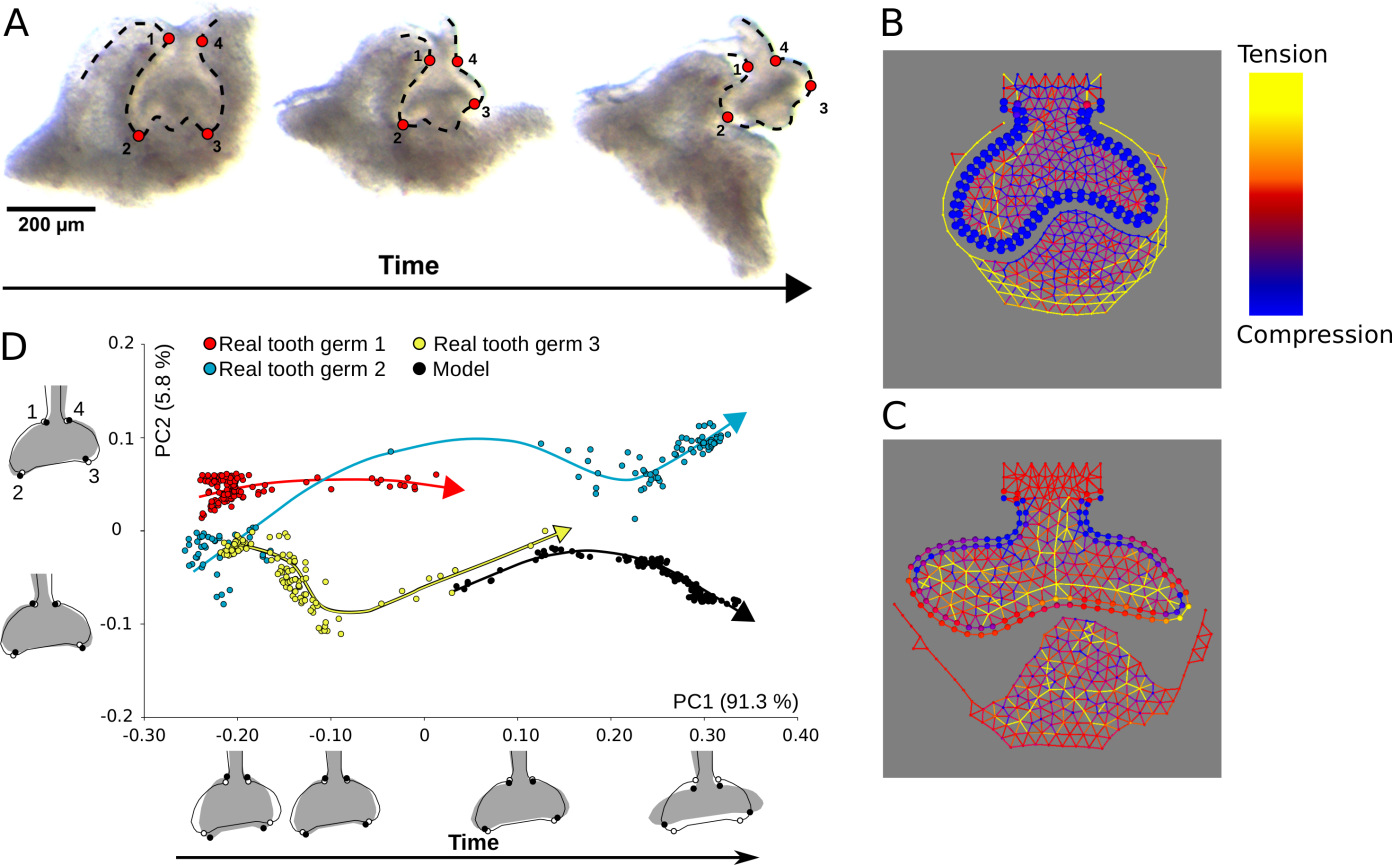
Epithelium-mesenchyme separation experiments. A, Pictures of the same E14.5 tooth germ before immersion in dispase with the four 2D configuration of landmarks used in Geometric Morphometrics analyses (left), 14 mins after (middle) and 40 minutes after (right). Landmark configurations were collected for each of the 120 time frames which compose the total duration of the separation experiment. B, Depiction of mechanical stresses in the *in silico* separation assay before separation (left) and C, after the tissues have reached mechanical equilibrium (right). Colour rods indicate direction and intensity (yellow for tension, blue for compression) at a certain cell-cell contact (as in Fig 7). Growth and adhesion parameters used: *s*_*epi*_ = 0.055, *s*_*sup*_ = 0.033, *s*_*mes*_ = 0.245, *b*_*ee*_ = 5.0, *b*_*es*_ = 5.0, *b*_*em*_ = 5.0, *b*_*ss*_ = 5.0, *b*_*mm*_ = 5.0. D, PCA of tooth germ variation during separation from experimental data from three different tooth germs (red, yellow and blue dots, n = 120 each), and from the simulation data shown in B (black dots, n = 120). The arrows point the flow of time for each dataset. The outline drawings of tooth germs show the patterns of shape changes associated with each PC from the overall average shape (solid grey outline with grey background and open circles) for different PC scores (solid black outline and solid black circles). Note that the outline drawings are depicted for an easier visualisation.

To test the model prediction, we performed an enzymatic separation of epithelium and mesenchyme on dissected E14.5 mouse first molars sliced in thick frontal sections (roughly 400 μm). Molar sections were submerged in dispase laying flat on the bottom of a glass dish so that one of the sliced surfaces was facing upwards. Dispase digests extracellular matrix, including the basement membrane and, given enough time, the epithelium and mesenchyme will separate from one another (S1 Video, also see [6]). The results show that the follicular mesenchyme on one side of the tooth germ peeled off from the epithelium, and eventually the whole mesenchyme recoiled towards the other side of the tooth germ (n = 6). At the same time, the cervical loops detached from the mesenchyme and rapidly changed their orientation, with the growth angle increasing to reach roughly 180° (Fig 9A). This reorientation of the cervical loops was unlikely to be due to growth since it occurred in less than 1 hour. No recoil in the mesenchyme was observed when the same experiment was performed on E13.5 tooth germs (bud stage, S8 Fig).

We then proceeded to reproduce the separation assay *in silico*. Since the tissue deformations essentially took place within a two-dimensional plane, we used the 2D version of the model. We first simulated tooth development until cap stage (Fig 9B), then we set the epithelial-mesenchymal adhesion to 0. We resumed the simulation until the system was at equilibrium again (i.e. no cell movement) (Fig 9C). We repeated the same procedure with different combinations of adhesion parameters (S9 Fig). We observed in all cases that the mesenchyme buccal and lingual to the tooth germ retracted towards the mid line of the tooth germ and in most cases the growth angle increased (Fig 9B and 9C, S9 Fig), in accordance with the experimental observations (Fig 9A). In order to quantify the model and experimental deformations, we tracked tissue deformation over time using specific morphological landmarks in the real tooth germs and in the model (Fig 9A–9C), and performed a principal components analysis on shape data extracted from these biological landmarks (real tooth germs n = 3, model tooth germs n = 1, Fig 9D, see Methods). The first principal component (PC) explained 91.3% of the total variation as an increase of the growth angle of the cervical loops (Fig 9D). Furthermore, time evolution both *ex vivo* and *in silico* consistently correlated with an increase in PC1 (Fig 9D, arrows).

By plotting the mechanical stresses over the tooth germ during the separation simulations we were able to obtain insights about the origins of the observed tissue deformations. The follicular mesenchyme retraction coincided in time, in the model, with a decrease in the tension between its cells suggesting that this tension may be also responsible for the recoil in the follicular mesenchyme in the *ex vivo* experiments (Fig 9B and 9C, S10 Fig). The reorientation of the epithelium and suprabasal layers coincided with an increase in tension in the suprabasal cells and a relaxation of the compression of the epithelial cells (Fig 9B and 9C, S10 Fig). This reorientation coincided with the arising in the suprabasal layer of an arch of tension along the bucco-lingual axis (Fig 9C, S10 Fig). In other words, a large part of the tension is distributed along a line of contiguous cells going from the buccal to lingual side of the suprabasal layer. By following the evolution of the mechanical stresses during the *in silico* separation, it can be seen that this arch was a result of the expansion of the epithelium that, by being mechanically attached to the suprabasal layer, pulled the suprabasal layer along the length of the cervical loops. The separation of the epithelium and the mesenchyme allows the suprabasal layer to relax the tension in this arch. As a result, the curvature and tension in this arch decrease and the cervical loops reorient in the bucco-lingual direction (S10 Fig).

## Discussion

### Cervical loops form by differential growth and adhesion

It has been argued that the compression of an epithelium (along its plane) will lead to its buckling (out of plane folding) (reviewed in [13]). The formation of small folds, or villi, in the small intestine, for example, has been proposed to occur by this mechanism [12]. In our model, the expansion of the growing epithelium is restricted by both the mesenchyme and the suprabasal layer. Suprabasal growth tends to push the epithelium outwards against the mesenchyme, but excessive growth will lead to a globular shape, reminiscent of an enlarged tooth bud (e.g. S11C Fig, left most column). On the contrary, as long as the suprabasal layer grows relatively slower than the epithelium, the latter will need to fold in order to keep contact with the former due to their heterotypic adhesion (e.g. S11C Fig, left most column). In other words, a buckled (cap) shape requires a higher surface (i.e. epithelium) to volume (i.e. suprabasal layer) ratio than a globular (bud) shape. The mesenchyme acts as a barrier to the outwards expansion of the epithelium, both due to the accumulated tension on the external layers and the pressure of the growing mesenchyme underneath the enamel knot. Even when high suprabasal growth is forcing the tooth germ into a globular shape, high mesenchymal growth can contribute to the epithelial folding by pushing the epithelium from underneath (e.g. S11C Fig, top row). For the same reason, high mesenchymal growth prevents epithelial buckling in the central part of the tooth germ and forces the epithelium to fold around the growing mesenchyme (Fig 3D), a process which also depends on the epithelial-mesenchymal heterotypic adhesion. Cervical loops, therefore, form only on the sides (buccal, lingual, anterior and posterior) of the dental mesenchyme.

### Differential adhesion regulates the orientation of the cervical loops

Our model predicts that three different factors regulate the orientation of the cervical loops.

1. The mesenchyme homotypic adhesion reduces the growth angle of the cervical loops. Growth and expansion of the epithelium and suprabasal layer stretch the outermost layers of follicular mesenchyme. In that sense, the follicular mesenchyme imposes a mechanical resistance to the expansion of epithelium and suprabasal layer. The strength of this resistance is proportional to the force required to separate mesenchymal cells from each other, which in turn depends on the mesenchymal homotypic adhesion (Fig 7A and 7B). As a result of this resistance, the growing cervical loops can not expand against the follicular mesenchyme and do so, instead, tangential to it (Fig 6A). In the absence of mesenchymal adhesion, the mesenchyme does not act as a barrier and the cervical loops grow in the bucco-lingual direction (Fig 6A, Fig 7C).
2. Suprabasal homotypic adhesion increases the growth angle of the cervical loops. Since the cervical loop epithelium grows faster than the suprabasal tissue on top of it, the latter is stretched in the direction of growth of the cervical loops. Higher values of suprabasal adhesion will result in stronger resistance to that stretching. Thus, tensions accumulate in the suprabasal layer along the length of the cervical loops (Fig 7B and 7C). Such tensions act as a cable connecting the tip of the cervical loop to the centre of the tooth germ from the inside that, when it is tensed, will tend to reduce the curvature of the cervical loops, thus orienting them in the bucco-lingual direction (Fig 7A and 7C).
3. Epithelial-mesenchymal adhesion regulates the downward growth of the cervical loops and the surrounding of the dental mesenchyme by the former (Fig 6B). When heterotypic adhesion is high, mesenchymal cells will tend to maximise the number of neighbouring epithelial cells. This is accomplished by the cervical loops folding downwards in order to surround the large mass of dental mesenchyme.

### The model predicts the asymmetry between the anterior-posterior and bucco-lingual cervical loops

This asymmetry can be understood from the geometry of the initial conditions (see S1 Appendix for more details). The early tooth bud *in vivo* and in the initial conditions of the model are both longer in the antero-posterior (AP) axis than in the bucco-lingual (BL) axis. That asymmetry was not observed when we simulated teeth whose initial conditions had radial symmetry (i.e. initial conditions are as long in the AP than in the BL axis) (Fig 8E–8H). Seen from below (i.e. from the dental mesenchyme), the initial epithelium looks like an ellipsoid and, as such, it has a higher curvature in the AP sides than in the BL sides (Fig 8A–8D), i.e. a higher ratio between surface and enclosed volume (S12A Fig). Because of that, the same amount of growth will lead to less elongation of the cervical loops in the AP than in the BL sides. We have devised a simplified geometrical argument showing that the elongation rate of the BL loops would follow an exponential growth function with exponent proportional to t (time), whereas the in the AP loops the exponent would be proportional to t/2 (S12B Fig, see details of the argument in S1 Appendix). The fact that this asymmetry was not so apparent when the mesenchymal homotypic adhesion or the epithelial-mesenchymal adhesion were very low is simply due to the fact that in these cases the cervical loops did not grow downwards anyway. Our model, thus, makes apparent that even when the tissues grow at the same rate everywhere, the AP vs. BL asymmetry of the initial conditions would inevitably lead to deeper lateral cervical loops than anterior and posterior loops.

### The model predicts patterns of mechanical stress in the different tooth germ tissues

The large recoil observed in the follicular mesenchyme after the experimental separation suggests that there is a line of tension running from buccal to lingual side of the tooth germ. A similar deformation was observed in the follicular mesenchyme in the *in silico* separation, although with less intensity.

The most visible deformation in the epithelium during the separation experiment is the reorientation of the cervical loops towards the bucco-lingual axis. The same kind of reorientation is observed in the *in silico* experiment. In the model, the reorientation of the cervical loops is due to the combination of two processes. One is the expansion of the epithelium that was under compression before the separation, and the other is the relaxation of the tension in the suprabasal layer. This latter tension is the result of the pull on the suprabasal layer by the expanding epithelium (as explained above). This latter tension is roughly distributed as an arch that will tend to flatten after the separation, thus reorienting the cervical loops in the bucco-lingual direction (S10 Fig). The combination of compression in the epithelium and tension in the suprabasal layer may also account for the epithelial deformation observed in the *ex vivo* separation. Our experiments, however, were not able to discern between stresses in the epithelium and the suprabasal layer.

In a recent study [22], a different kind of mechanical perturbation was performed at the placode stage of tooth (E12.5), i.e. before the tooth bud forms. Using thick frontal slices of tooth germs, Panouspopoulou and Green performed a cut in the oral epithelium at one side of the placode and observed that the epithelium by the side of the placode recoiled towards the mid line. They also interpreted this recoil as a consequence of a bucco-lingually oriented tension, in this case located in the suprabasal layer of the tooth placode. Along these lines we have observed that, when the separation experiment is performed on bud stage tooth germs (E13.5) the mesenchyme did not recoil (S8 Fig), indicating that the tension in the mesenchyme may build up between the bud and cap stages, as our model predicts (S13 Fig).

### Role of differential growth and adhesion in other ectodermal organs

In this study we have explored, theoretically and experimentally, the role differential growth and adhesion on the transition from the bud stage, common to several ectodermal organs, to the specific cap shape of the early tooth germ. Our model simulates the formation of the tooth germ by combining the aforementioned processes, and accounting for cell and tissue mechanical interactions that result in tooth specific shape transformations. Even though our model accounts only for early tooth morphogenesis, it does so by implementing a set of cell and mechanical processes common to the ensemble of ectodermal organs. Thus, the dynamics produced by this model leading to early tooth-specific shapes (or failing to do so), may shed light on how other ectodermal organs undergo their specific transformations after bud stage.

For instance, some of the model dynamics may also apply to the formation of epithelial folds surrounding a mesenchymal condensate in the hair follicle after its bud stage [23]. In a homologous fashion, the formation of these folds may be a result of increased epithelial growth relative to suprabasal growth, whereas a high epithelial-mesenchymal adhesion may account for the surrounding of the mesenchyme by these folds. In another type of ectodermal organs, such as mammary, salivary glands and lungs, the epithelium folds in order to form branched structures, but never surrounds the mesenchyme [24]. *Ex vivo* and *in silico* studies of mouse lung epithelium suggest that mechanical buckling of the epithelium due to intrinsic growth plus mechanical interactions with the surrounding extracellular matrix is sufficient to account for branch formation [25]. In addition, it has been shown that the lung mesenchyme also contributes to epithelial folding by mechanically constricting the lung epithelium during branch formation [26]. Future studies should address whether differential tissue growth and mechanical interactions between epithelium and mesenchyme observed in the development of different ectodermal organs are regulated by a conserved developmental mechanism.

### Migration

Our model does not consider active cell migration or cell contraction (although EmbryoMaker can implement these cell processes). Active cell migration over an extracellular matrix substrate and cell intercalation have been shown to generate tissue-scale mechanical stresses [27, 28]. It has been shown that mesenchymal cells at the early bud stage actively migrate towards the source of an FGF8 gradient in the bud epithelium [29]. It has also been shown at the tooth placode stage that perturbation of the Shh pathway altered the width and depth of the placode, suggesting the presence of cell intercalation in the suprabasal layer [22]. Morita and collaborators also argued that active migration mediated by high F-actin turnover and the LIMK-cofilin pathway is present in the growing regions of the tooth germ epithelium [6]. Even though we acknowledge that active cell migration and cell intercalation may have a role in tooth development and, perhaps, would improve our model if included, we show that several features of tooth development related to tissue growth, cell movement, and tissue mechanics can already be explained by considering only passive cell movement resulting from cell adhesion and proliferation.

### Concluding remarks

Our new mathematical model of tooth development provides detailed quantitative explanations on how biomechanical processes may drive tooth germ morphology to change the specific way it does in 3D space and over developmental time. This included explanations on how morphology will change in specific ways when these biomechanical processes are altered and, thus, understanding not only the wild-type but also its variational properties. To our knowledge no such explanations have been provided for any ectodermal organ, although they are well studied in other aspects of their development. In spite of the increase in complexity of tooth germ morphology during development, two biomechanical processes seem enough to explain it. This result highlights how the combination of experimental results with computational models of biomechanical processes can help providing relatively simple explanations for seemingly complex processes such as the development of morphology and its variation.

## Materials and methods

### Ethics statement

All animal work was conducted accordingly to the guidelines required by the Finnish authorities (ESAVI-2984-04.10.07–2014, KEK13-020). Mouse specimens were sacrificed by anaesthetising with CO_2_ first followed by cervical dislocation.

### Model design

The following section describes the basics of EmbryoMaker and how we build the tooth-specific model based on it (see [15] for a more extensive description).

Mesenchymal and suprabasal cells are made of spherical bodies, that we call nodes, whereas epithelial cells are made of cylindrical bodies consisting of two nodes (one basal and one apical bound by an elastic link) (S1A and S1B Fig). The movement of nodes follows an overdamped Langevin equation of motion,

$$\frac{\partial {\vec{r}}_{i}}{\partial t}=\sum_{j=1}^{{\mathrm{j=n}}_{v}}f_{\mathrm{Aij}}{\hat{u}}_{\mathrm{ij}}$$ (1)

where *r*_*i*_ is the position vector of node *i*, *n*_*v*_ is the number of nodes in the neighborhood of node *i*, *t* is time, *f*_*Aij*_ is the modulus of the force acting between node *i* and *j* and *u*_*ij*_ is the unit vector connecting *i* and *j* (see S1A–S1D Fig). The modulus and sign of the force is dependent on the distance between the two nodes,

$$\{\begin{array}{c} f_{\mathrm{Aij}}=\left(p_{i}^{\mathrm{REC}}{+p}_{j}^{\mathrm{REC}}\right)\left(d_{\mathrm{ij}}-\left(p_{i}^{\mathrm{EQD}}{+p}_{j}^{\mathrm{EQD}}\right)\right)\;\mathrm{if}\;d_{\mathrm{ij}}<\left(p_{i}^{\mathrm{EQD}}{+p}_{j}^{\mathrm{EQD}}\right)\\ f_{\mathrm{Aij}}{=k}_{\mathrm{ij}}^{\mathrm{ADH}}\left(d_{\mathrm{ij}}-\left(p_{i}^{\mathrm{EQD}}{+p}_{j}^{\mathrm{EQD}}\right)\right)\;\mathrm{if}\left(p_{i}^{\mathrm{EQD}}{+p}_{j}^{\mathrm{EQD}}\right)\leq d_{\mathrm{ij}}\leq \left(p_{i}^{\mathrm{ADD}}{+p}_{j}^{\mathrm{ADD}}\right)\\ f_{\mathrm{Aij}}=0\;\mathrm{if}\left(p_{i}^{\mathrm{ADD}}{+p}_{j}^{\mathrm{ADD}}\right){>d}_{\mathrm{ij}} \end{array}$$ (2)

When the distance between nodes *i* and *j* (*d*_*ij*_) is shorter than the sum of their radii at equilibrium (*p*^*EQD*^), there is a repulsive force proportional to the sum of the *p*^*REC*^ of each node (this coefficient determines their incompressibility). When this distance is longer than the equilibrium distance but shorter than the sum of the maximum radii of *i* and *j* (*p*^*ADD*^), there is an attractive force between nodes i and j. This force is proportional to *k*_*ij*_^*ADH*^,

$$k_{\mathrm{ij}}^{\mathrm{ADH}}=g_{\mathrm{im}}g_{\mathrm{jn}}b_{\mathrm{mn}}$$ (3)

where *g*_*im*_ is the amount of adhesion molecule *m* expressed in node *i* and *b*_*mn*_ is the adhesive affinity between adhesion molecules *m* and *n*.

The direction of force vectors differ between mesenchymal-mesenchymal, epithelial-epithelial and the epithelial-mesenchymal node interactions, since vectors need to be normal to the contact interface between nodes and nodes have different shapes in epithelial cells and mesenchymal cells (see [15] for a detailed explanation). The apical and basal nodes of epithelial cells are connected by an elastic spring that opposes any departure from an equilibrium distance between the apical and basal nodes of each cylinder [15]. The force generated by the spring is calculated as follows,

$${\vec{f}}_{\mathrm{Sij}}=k_{ij}^{\mathrm{HOO}}\left(d_{\mathrm{ij}}-p_{ij}^{\mathrm{EQS}}\right){\hat{s}}_{\mathrm{ik}}$$ (4)

where *k*_*ij*_^*HOO*^ *= p*_*i*_^*HOO*^ *+ p*_*j*_^*HOO*^ is the elastic coefficient of the spring (which is determined by the sum of the mechanical parameter *p*^*HOO*^ in both nodes), *d*_*ij*_ is the distance between node *i* and *j*, *p*_*ij*_^*EQS*^ is the equilibrium length of the spring between node *i* and *j* and *s*_*ik*_ is the unit vector connecting the two epithelial nodes.

Two additional force components are required in epithelial cells in order for them to organise as one layered sheets [15]. A radial force acts along the apical-basal axis of the cell and tends to restore displacements in that axis in respect to neighbouring cells in the epithelium, whereas a rotational force acts tangential to surface of the epithelium and tends to orient the apical-basal axis of cells normal to the epithelial plane. These forces are calculated as follows,

$$\vec{f_{ESTij}}=k_{ij}^{EST}\frac{\vec{m_{ijkl}}\cdot \vec{c_{ij}}}{\left|\vec{m_{ijkl}}\right|}\hat{m_{ijkl}}$$ (5)

$$\vec{f_{ERPij}}=p_{i}^{ERP}\frac{\vec{s_{ik}}\cdot \vec{c_{ij}}}{\left|\vec{s_{ik}}\right|}\hat{c_{ij}}$$ (6)

where *f*_*ij*_^*EST*^ is the radial bending force and *f*_*ij*_^*ERP*^ is the rotational bending force. We define *c*_*ij*_ as the vector connecting neighboring node *i* and *j*, *s*_*ik*_ and *s*_*jl*_ as the vectors that connect each apical node to their basal counterparts and *m*_*ijkl*_ as the sum of *s*_*ik*_ and *s*_*jl*_ which defines the vector normal to the apical or basal surface between *i* and *j*. The radial bending force always acts on the direction of *m*_*ijkl*_, and is proportional to the deviation of the angle formed by *m*_*ijkl*_ and *c*_*ij*_ from 90°. *k*_*ij*_^*EST*^ is the sum of the mechanical parameter *p*^*EST*^ of nodes *i* and *j* (see [15] for a detailed explanation). The rotational bending force is proportional to the deviation of the angle formed by *s*_*ik*_ and *c*_*ij*_ from 90°, but in this case the direction of the force is parallel to *c*_*ij*_, thus promoting a tilting of the epithelial cylinder that reaches an equilibrium (that is the force modulus becomes 0) when the apical-basal axis of the epithelial cylinder is normal to the apical/basal cell surface. *k*_*ij*_^*ERP*^ is the sum of the mechanical parameter *p*^*ERP*^ of nodes *i* and *j* (see [15] for a detailed explanation).

In summary, thus, the forces acting on an epithelial node are:

$$\frac{\partial {\vec{r}}_{i}}{\partial t}={\vec{f}}_{\mathrm{Sik}}+\sum_{j=1}^{n_{d}}\left(f_{\mathrm{Aij}}{\hat{u}}_{\mathrm{ij}}+{\vec{f}}_{\mathrm{ESTij}}+{\vec{f}}_{\mathrm{ERPij}}\right)$$ (7)

where *k* is the node in the same cylinder than *i* and the sum is made over all the neighboring nodes except for *k*.

Cell division is implemented by placing a new cell in a random position close to the old one. EmbryoMaker implements many other cell behaviors (such as apoptosis, cell contraction, extracellular secretion, cell growth, cell polarization) as rules acting on these nodes but we do not explain them because they are not used in the tooth-specific model.

Different gene products can be specified as transcription factors, adhesion molecules, extracellular diffusible signals, or receptors (see S1 Appendix). They can also be specified to regulate different cell behaviours, such as cell division. Each cell has a variable, *P*^*PHA*^, indicating the progression of the cell cycle (from 0 to 1). When *P*^*PHA*^ reaches 1, the cell divides.

### *Ex vivo* separation experiment

Molar germs were dissected from NMRI mice and sliced into thick frontal slices using fine tungsten needles. Molar sections were submerged on a glass Petri dish containing a dispase II solution, laying flat on the bottom of the dish. A small amount of DNAseI was added to the enzymatic solution in order to reduce the viscosity of the medium and facilitate tissue separation. A time-lapse sequence of the tooth sections in the solution was recorded for about 1 hour and a half in a Olympus SZX9 microscope. The tissues usually started to separate from each other about 25 minutes after being submerged in the solution, and it usually took about 40 minutes for the whole deformation to take place.

### Geometric morphometric analyses of tooth germs

For each tooth germ (n = 3) and the model (n = 1), 120 pictures that correspond to every time frame from the total duration of the separation experiment were taken (total = 480 pictures) at the same magnification. For each picture, a suite of 4 landmarks was collected in two dimensions using the freely available software tpsDig version 2.17 [30] to precisely characterize the architecture of tooth germs (Fig 9A) and was digitized twice to assess measurement error due to digitizing (total = 960 landmark configurations). Landmarks were located at the maximum of curvature at the junction between outer enamel epithelium and oral epithelium (landmarks 1 and 4) and at the maximum of curvature of the cervical loops (landmarks 2 and 3). From these 960 landmark configurations, shape was extracted by removing extraneous information of size, position and orientation via a Generalized Procrustes Analysis (GPA; e.g. [31]). A principal components analysis (PCA) was performed on these shape data to detect and localize very fine morphological variation in relation to the geometry of the tooth germs (Fig 9D). A one-way Procrustes analysis of variance (ANOVA) and a one-way ANOVA was used with centroid size (i.e., square root of the sum of the squared distances of all landmarks from their centroid (e.g. [32]) and decompose the total shape variation according to the main effect of ‘tooth germ’ (i.e. variation among tooth germs) and measurement error due to digitising in shape and size (e.g. [33]). The ANOVAs for both size and shape reveal that the main effect of ‘tooth germ’ is highly significant (*P* < 0.0001) compared to digitizing error, meaning that digitizing error in placing landmarks on tooth germs is highly negligible (S1 Table). Consequently, averages of both digitizing sessions were calculated to obtain a single configuration of landmarks per picture and these 480 mean shapes were used in all subsequent analyses. All analyses were carried out using the freely available software MorphoJ [34].

## Supporting information

S1 Fig Implementation of cell-cell mechanical interactions in the EmbryoMaker framework (Marin-Riera et al. 2016). A, Mechanical interactions between spherical nodes (mesenchyme and suprabasal layer) are determined by the distance between their centers and their equilibrium radius (edge of the darker circle). B–D, Mechanical interactions between two epithelial cells or between an epithelial cell and a spherical node act along a vector normal to the surface of contact between the two elements. E, The two nodes composing an epithelial cell are tied by an unbreakable elastic spring. F, Epithelial bending is regulated by two different forces, a bending radial force (left) and a bending rotational force (right). All arrows represent force vectors.
(PNG)

S2 Fig Parameter exploration of different cell mechanical parameters. A parameter exploration was performed by varying the cell incompressibility parameter (*p*^*REC*^) independently for epithelial, suprabasal and mesenchymal cells. A-C show shape variation for different combinations of suprabasal and mesenchymal values of *p*^*REC*^, keeping epithelial *p*^*REC*^ constant at low (A), intermediate (B) and high (C) values. Higher values of *p*^*REC*^ within a tissue lead to a higher resistance to compression and a higher volume occupied relative to the other tissues. D, a different parameter exploration was performed by varying the two parameters controlling epithelial bending forces (*p*^*EST*^ and *p*^*ERP*^). Null values of either of these parameters lead to breakdown of epithelium, whereas high values of *p*^*EST*^ lead to cervical loops that are slightly more straight.
(PNG)

S3 Fig Parameter screening of the tissue specific proliferation rates (*s*_*epi*_, *s*_*sup*_ and *s*_*mes*_). A, B, C, Variation in tooth germ morphology with different combinations of *s*_*epi*_ and *s*_*sup*_, keeping *s*_*mes*_ constant, under the different hypotheses (frontal sections depicted). In all cases, cervical loops form when *s*_*epi*_ is relatively high and *s*_*sup*_ is relatively low, but only in hypotheses II and III these are oriented downwards as in tooth development. D, Variation in tooth morphology when *s*_*mes*_ is changed under hypothesis II. E, Same as in D for hypothesis III. A relatively high mesenchymal proliferation is necessary for the proper formation of the tooth crown. The epithelium is shown in blue, the enamel knot is shown in red, the mesenchyme in purple and the suprabasal layer in yellow. *s*_*epi*_, *s*_*sup*_ and *s*_*mes*_ values are expressed in *h*^*-1*^.
(PNG)

S4 Fig Growth curve measurements for empirical and *in silico* data. A, B, individual frames from the time-lapse video (taken from Morita et al. 2016 with permission) that we chose as starting and finishing time points for the measurement. The white segment in A corresponds to the reference unit of length we chose for measuring epithelial perimeter and suprabasal surface area. C, Depiction of how we took the measurements in the empirical data set. A polygon was drawn by hand on the epithelial-suprabasal interface (cyan and red segments) using ImageJ (Schindelin et al. 2012). The surface area of the suprabasal tissue was calculated from the polygon’s surface area, whereas the length of the epithelium was calculated from the total length of the cyan segment. D, depiction of the 2D model initial conditions, showing the length of the reference unit used for the model measurements (white segment). E, depiction of how the measurements were taken on the 2D model. The suprabasal tissue surface area and epithelial perimeter were calculated using a Delaunay triangulation. The polygon depicted in cyan and red marks the limit of the suprabasal tissue used for the surface area calculations and the cyan segment was used to calculate the length of the epithelium.
(PNG)

S5 Fig Sensitivity analysis of the 2D model parameter screening. Each plots shows the distribution of the standard error between model and empirical data in log scale (Y axis) separately for the different values of one specific parameter (X axis) as a violin plot. Each violin groups simulation runs with a fixed value of a specific parameter, while the rest may have different values. The width of the “violin” at a certain height indicates the density of data points (i.e. simulation runs) that show a specific value of standard error. The analysis was done for hypotheses II (A-D) and III (B-H). A, E, violin plots for the different values of parameter *s*_*epi*_. B, F, violin plots for the different values of parameter *s*_*sup*_. C, G, violin plots for the different values of parameter *s*_*mes*_. D, H, violin plots for the different values of the 5 adhesion parameters. The standard error shows the greatest variation across different values of *s*_*epi*_, whereas in shows the least variation across values of *s*_*mes*_ and the adhesion parameters. The violin plots were made with the vioplot R package.
(PNG)

S6 Fig Parameter exploration of differential adhesion parameters. A, Tooth germ morphologies are shown with different combinations of mesenchymal homotypic adhesion and suprabasal homotypic adhesion values, while the other adhesion parameters are set to 1.0. High values of mesenchymal homotypic adhesion result in low growth angles whereas high values of suprabasal homotypic adhesion result in high growth angles. B, Morphologies are shown with different combinations of mesenchymal homotypic adhesion and epithelial-mesenchymal adhesion values (other adhesion parameters set to 1.0). High values of epithelial-mesenchymal adhesion result in low growth angles. Frontal sections shown. Epithelium in blue, suprabasal layer in yellow, mesenchyme in purple and enamel knot in red. C, D, Sagital sections of the same morphologies depicted in A and B respectively. Variation in adhesion parameters has the same effect in the anterior and posterior loops compared to the buccal and lingual, albeit the former tend to be shorter. Epithelium in yellow, suprabasal layer in blue, mesenchyme not shown. E, F, Same morphologies depicted in A and B respectively, only showing the epithelium and seen from below. The colour code indicates the heigth, or position in the Z axis. All the simulations in this screening used the following growth parameters: *s*_*epi*_ = 0.063, *s*_*sup*_ = 0.021, *s*_*mes*_ = 0.182.
(PNG)

S7 Fig Depiction of forces at the interface between epithelium and suprabasal layer and between epithelium and mesenchyme. Epithelial cells are depicted as nodes (small spheres) connected by a spring (green). Thin frontal sections are shown in order to see clearly individual rods (i.e. force mechanical interactions. Parameters used in A, b_ee_ = 1.0, b_es_ = 1.0, b_em_ = 1.0, b_ss_ = 5.0, b_mm_ = 1.0. Parameters used in B, b_ee_ = 1.0, b_es_ = 5.0, b_em_ = 5.0, b_ss_ = 5.0, b_mm_ = 1.0. Colour coding is the same as in Fig 7.
(PNG)

S8 Fig Separation experiment on an E13.5 tooth bud. No major deformation in the epithelial bud is observed, and neither in the surrounding mesenchyme. Dashed line shows the epithelial mesenchymal boundary. Time after immersion in the dispase solution is shown beneath each panel. Scale bar is 200 μm long.
(PNG)

S9 Fig *In silico* separation experiments performed with different adhesion parameters. A, B, cap stage tooth germs simulated with different adhesion parameters. C, D, the same tooth germs as in A and B respectively, after the separation. Growth parameters used: *s*_*epi*_ = 0.055, *s*_*sup*_ = 0.033, *s*_*mes*_ = 0.245.
(PNG)

S10 Fig Time series of the *in silico* separation shown on Fig 9, depicting mechanical forces at different time points. Colour code indicates mechanical stress, the same as in Figs 7 and 9.
(PNG)

S11 Fig The whole growth parameter exploration for hypothesis III in the 3D model. Each panel (A-D) displays model simulations for all the permutations of suprabasal and mesenchymal growth rate values (*s*_*sup*_ and *s*_*mes*_) for a constant value of epithelial growth rate (*s*_*epi*_). *s*_*epi*_ values gradually increase in each subsequent panel. Colouring as in Fig 3.
(PNG)

S12 Fig Depiction of the geometric argument explaining differences in length between AP and BL cervical loops. A, depiction of a simplified 2D tooth germ, seen from below. These could also be seen as tooth germs in which the angle of growth is 180 degrees. The light shade of blue indicates the anterior (A) and posterior (P) parts of the germ, where the A and P cervical loops are growing. The dark blue shade indicates the buccal (B) and lingual (L) portions of the tooth germ, where the B and L cervical loops are growing. B, the length of the cervical loops in the AP side (*r*_*AP*_) corresponds to the radius of a circle segment, whereas in the BL side (*r*_*BL*_) it corresponds to the side of a rectangle. Assuming that uniform tissue growth leads to an increase in surface area at a constant rate both in the AP and BL parts (*S*_*AP*_ and *S*_*BL*_), the resulting elongation rates for the AP and BL cervical loops follow an exponential function with an exponent of *t/2* and *t* respectively (*t* corresponds to time).
(PNG)

S13 Fig Time series of the three simulations (A-C) shown in Fig 7A–7C, depicting mechanical forces on the mesenchyme (top series for each panel) and the suprabasal layer (bottom series for each panel) at different time points. Top series for each simulation shows only the tooth mesenchyme through a sagittal cut (only the buccal half is displayed). Bottom series for each simulation shows only the suprabasal layer seen from below (as in Fig 7, right column). Rods follow the same colour coding as in Fig 7 (yellow for tension, blue for compression, red for null force). Left most pictures show the final morphology that corresponds to the ones displayed in Fig 7.
(PNG)

S1 Table Analyses of variance (ANOVAs) for landmark digitizing error in shape and size (i.e. centroid size) for tooth germs and the model. SS, sum of squares; MS, mean square; Df, degrees of freedom; F, F-value; *P*, *P* value.
(PDF)

S1 Video Time lapse recording of one separation experiment of six E14.5 tooth germs. Tooth germs were dissected and sectioned into thick slices. Sections were submerged into a dispase solution with the sectioned surface laying flat on the bottom. It can be seen that, as soon as epithelium and mesenchyme detach, the mesenchyme recoils towards one side of the tooth germ and the cervical loops in the epithelium change orientation. The real length of the recording is 40 minutes, each frame was taken every 20 seconds.
(AVI)

S1 Appendix Additional information on model design and Methods. (PDF)
