# Supplementary figures and images for: Differential tissue growth and cell adhesion alone drive early tooth morphogenesis: An ex vivo and in silico study
Source: PLoS Comput Biol. 2018 Feb 26;14(2):e1005981. doi: 10.1371/journal.pcbi.1005981 (PMC5843354; doi:10.1371/journal.pcbi.1005981)

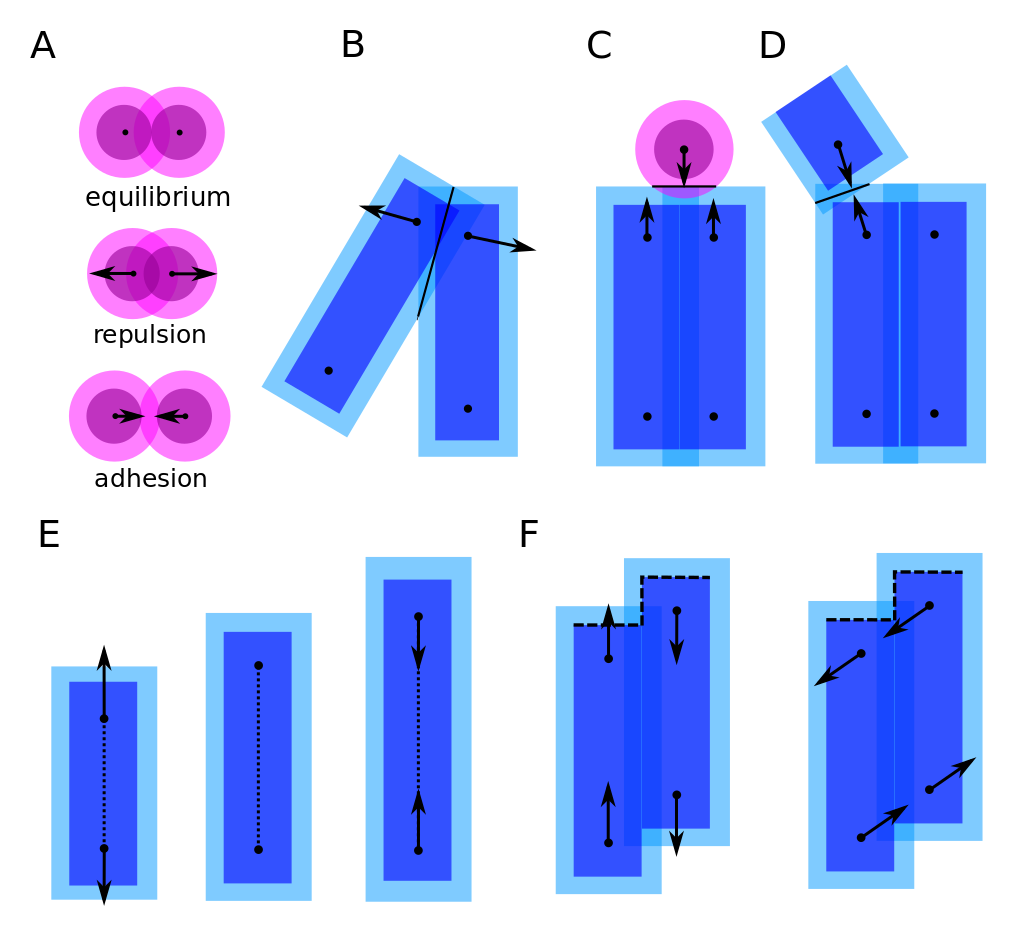

Supplement: S1 Fig — A, Mechanical interactions between spherical nodes (mesenchyme and suprabasal layer) are determined by the distance between their centers and their equilibrium radius (edge of the darker circle). B–D, Mechanical interactions between two epithelial cells or between an epithelial cell and a spherical node act along a vector normal to the surface of contact between the two elements. E, The two nodes composing an epithelial cell are tied by an unbreakable elastic spring. F, Epithelial bending is regulated by two different forces, a bending radial force (left) and a bending rotational force (right). All arrows represent force vectors. (PNG) [file pcbi.1005981.s001.png]

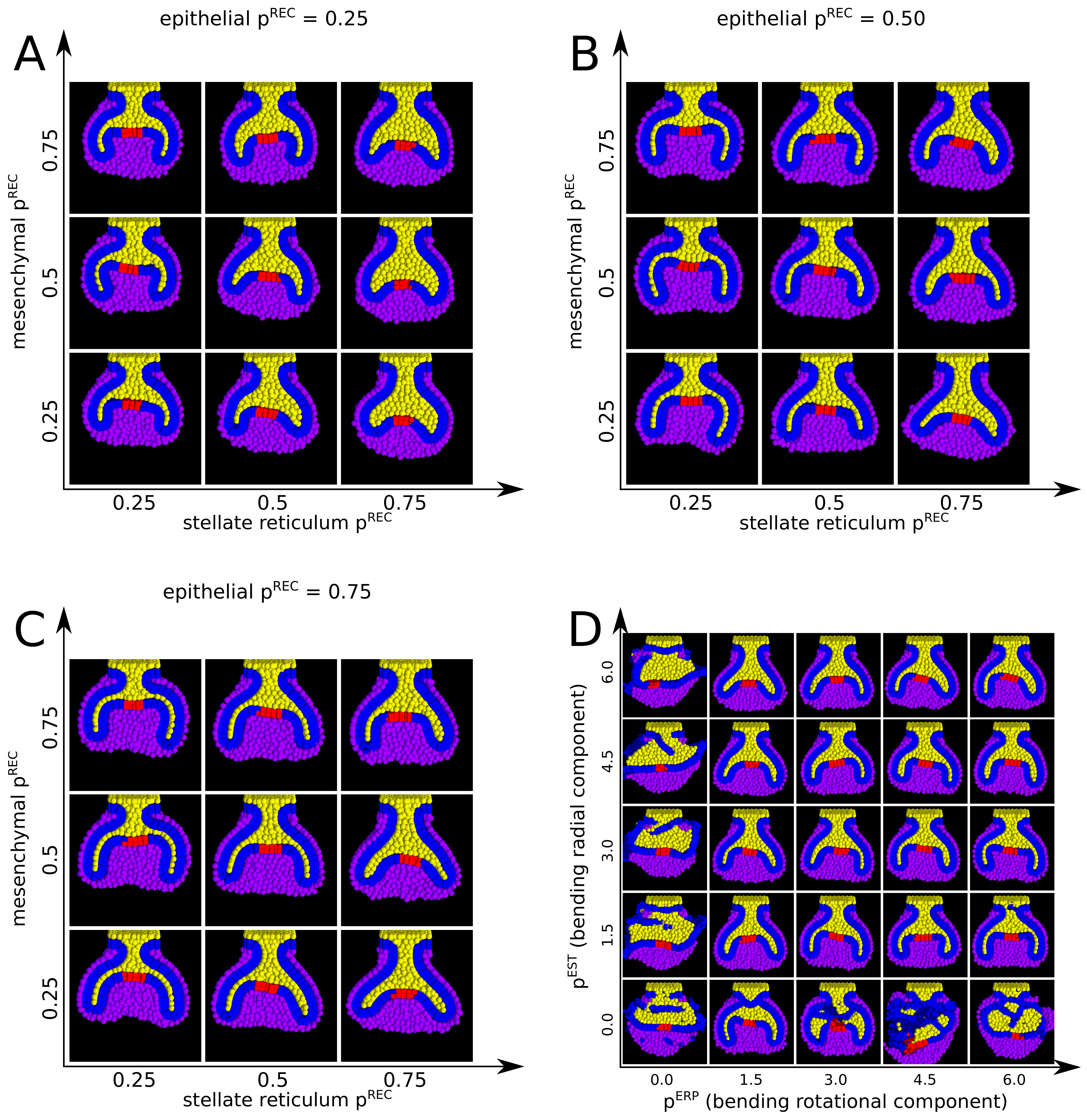

Supplement: S2 Fig — A parameter exploration was performed by varying the cell incompressibility parameter (pREC) independently for epithelial, suprabasal and mesenchymal cells. A-C show shape variation for different combinations of suprabasal and mesenchymal values of pREC, keeping epithelial pREC constant at low (A), intermediate (B) and high (C) values. Higher values of pREC within a tissue lead to a higher resistance to compression and a higher volume occupied relative to the other tissues. D, a different parameter exploration was performed by varying the two parameters controlling epithelial bending forces (pEST and pERP). Null values of either of these parameters lead to breakdown of epithelium, whereas high values of pEST lead to cervical loops that are slightly more straight. (PNG) [file pcbi.1005981.s002.png]

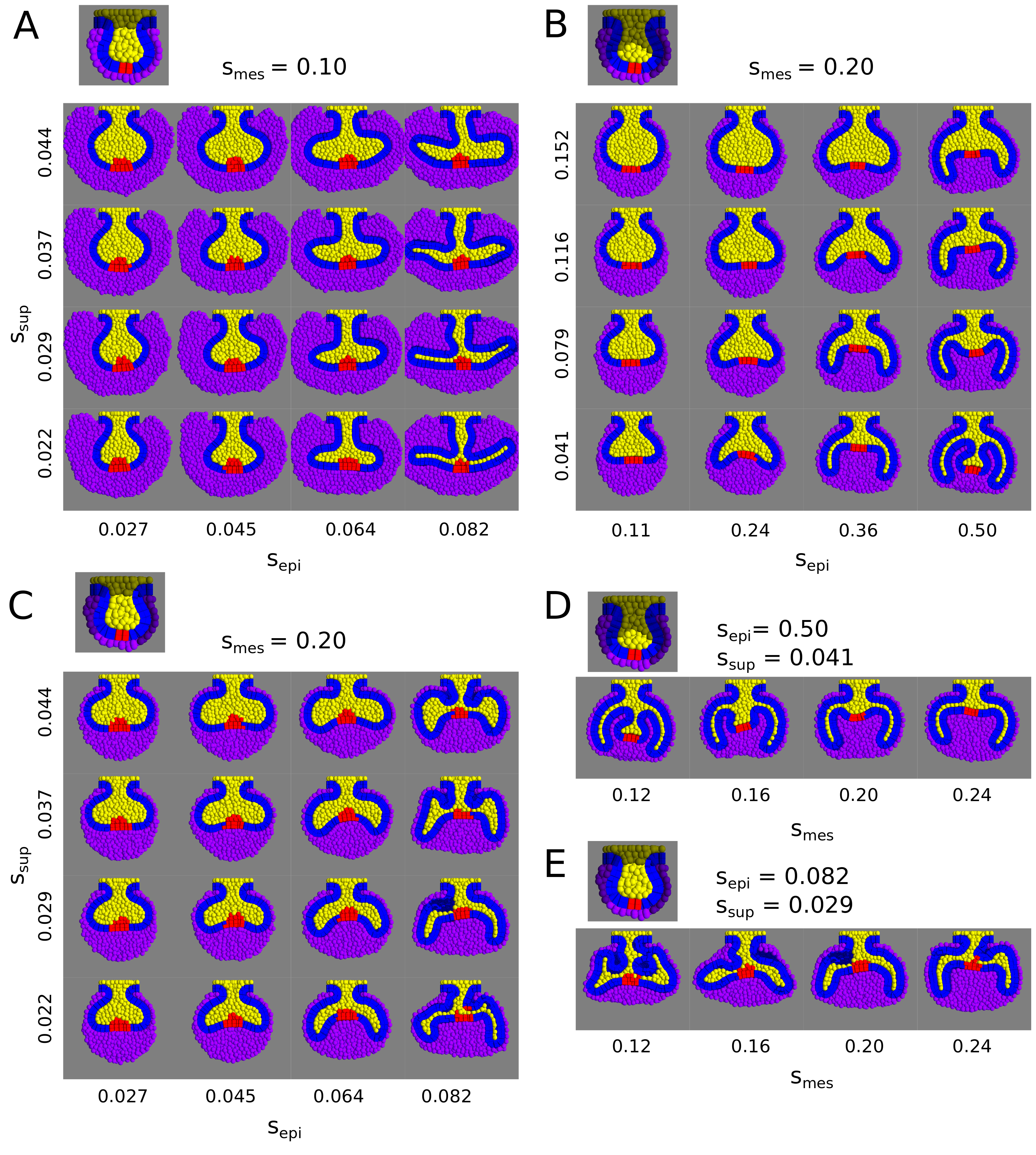

Supplement: S3 Fig — A, B, C, Variation in tooth germ morphology with different combinations of sepi and ssup, keeping smes constant, under the different hypotheses (frontal sections depicted). In all cases, cervical loops form when sepi is relatively high and ssup is relatively low, but only in hypotheses II and III these are oriented downwards as in tooth development. D, Variation in tooth morphology when smes is changed under hypothesis II. E, Same as in D for hypothesis III. A relatively high mesenchymal proliferation is necessary for the proper formation of the tooth crown. The epithelium is shown in blue, the enamel knot is shown in red, the mesenchyme in purple and the suprabasal layer in yellow. sepi, ssup and smes values are expressed in h-1. (PNG) [file pcbi.1005981.s003.png]

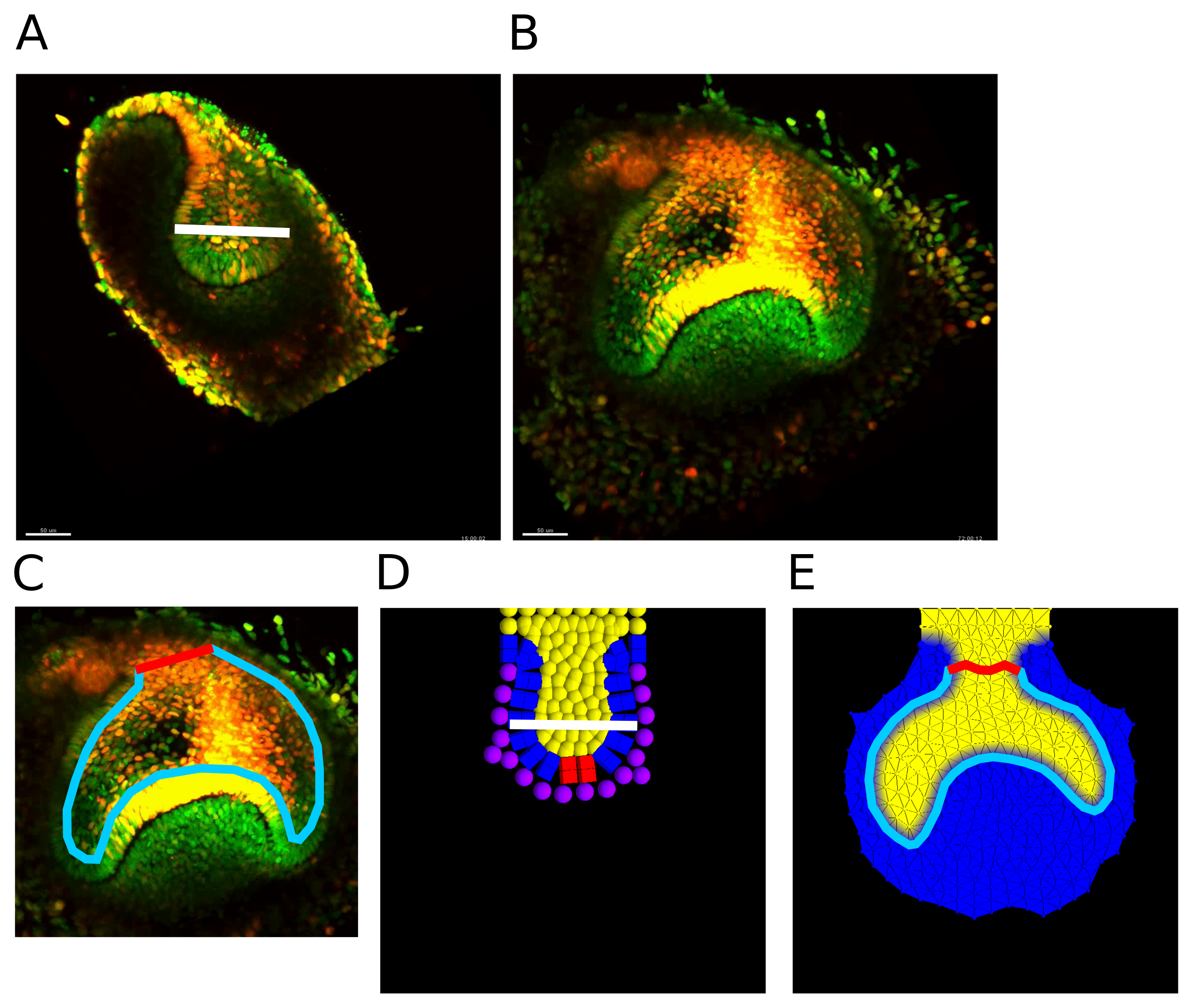

Supplement: S4 Fig — A, B, individual frames from the time-lapse video (taken from Morita et al. 2016 with permission) that we chose as starting and finishing time points for the measurement. The white segment in A corresponds to the reference unit of length we chose for measuring epithelial perimeter and suprabasal surface area. C, Depiction of how we took the measurements in the empirical data set. A polygon was drawn by hand on the epithelial-suprabasal interface (cyan and red segments) using ImageJ (Schindelin et al. 2012). The surface area of the suprabasal tissue was calculated from the polygon’s surface area, whereas the length of the epithelium was calculated from the total length of the cyan segment. D, depiction of the 2D model initial conditions, showing the length of the reference unit used for the model measurements (white segment). E, depiction of how the measurements were taken on the 2D model. The suprabasal tissue surface area and epithelial perimeter were calculated using a Delaunay triangulation. The polygon depicted in cyan and red marks the limit of the suprabasal tissue used for the surface area calculations and the cyan segment was used to calculate the length of the epithelium. (PNG) [file pcbi.1005981.s004.png]

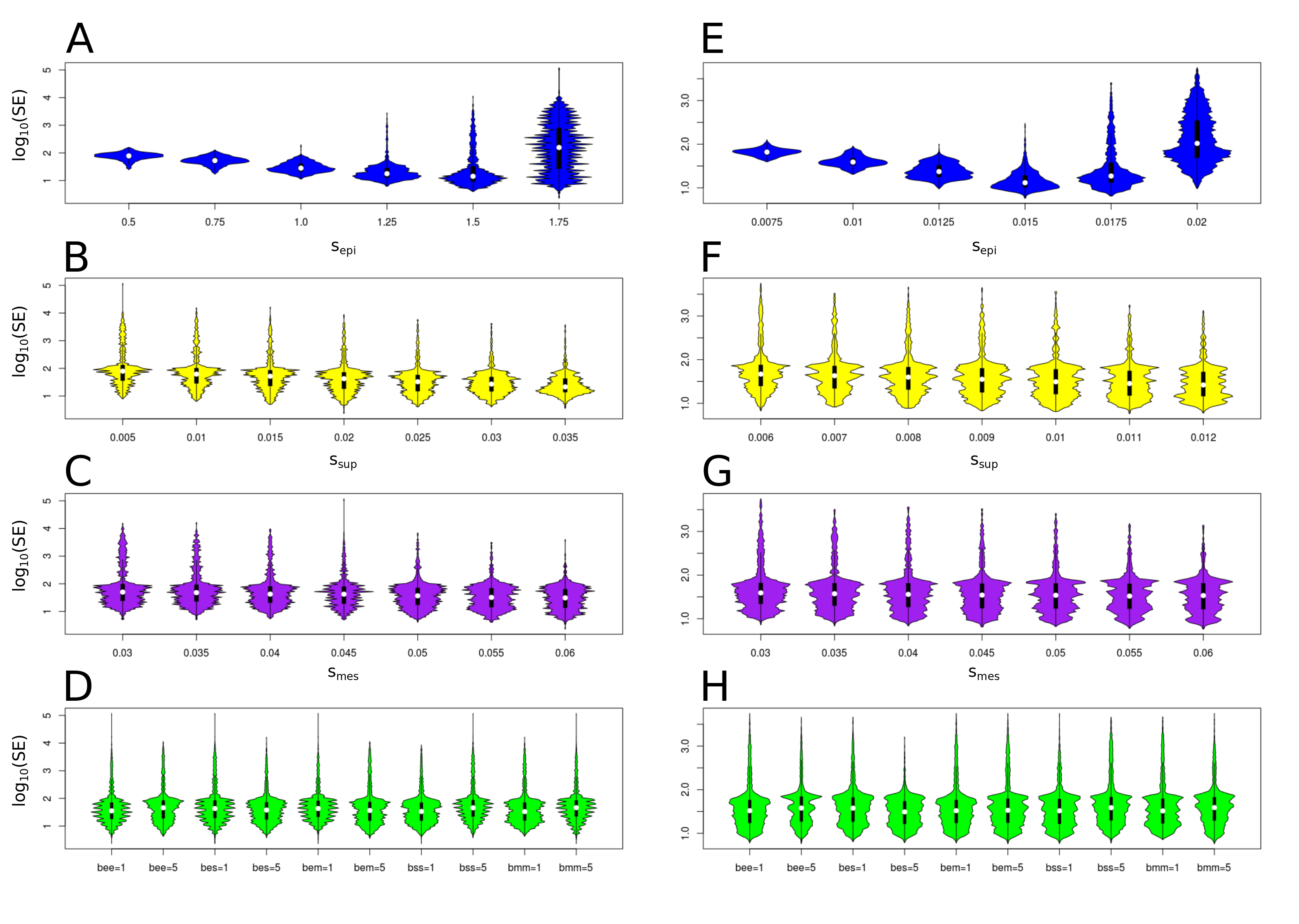

Supplement: S5 Fig — Each plots shows the distribution of the standard error between model and empirical data in log scale (Y axis) separately for the different values of one specific parameter (X axis) as a violin plot. Each violin groups simulation runs with a fixed value of a specific parameter, while the rest may have different values. The width of the “violin” at a certain height indicates the density of data points (i.e. simulation runs) that show a specific value of standard error. The analysis was done for hypotheses II (A-D) and III (B-H). A, E, violin plots for the different values of parameter sepi. B, F, violin plots for the different values of parameter ssup. C, G, violin plots for the different values of parameter smes. D, H, violin plots for the different values of the 5 adhesion parameters. The standard error shows the greatest variation across different values of sepi, whereas in shows the least variation across values of smes and the adhesion parameters. The violin plots were made with the vioplot R package. (PNG) [file pcbi.1005981.s005.png]

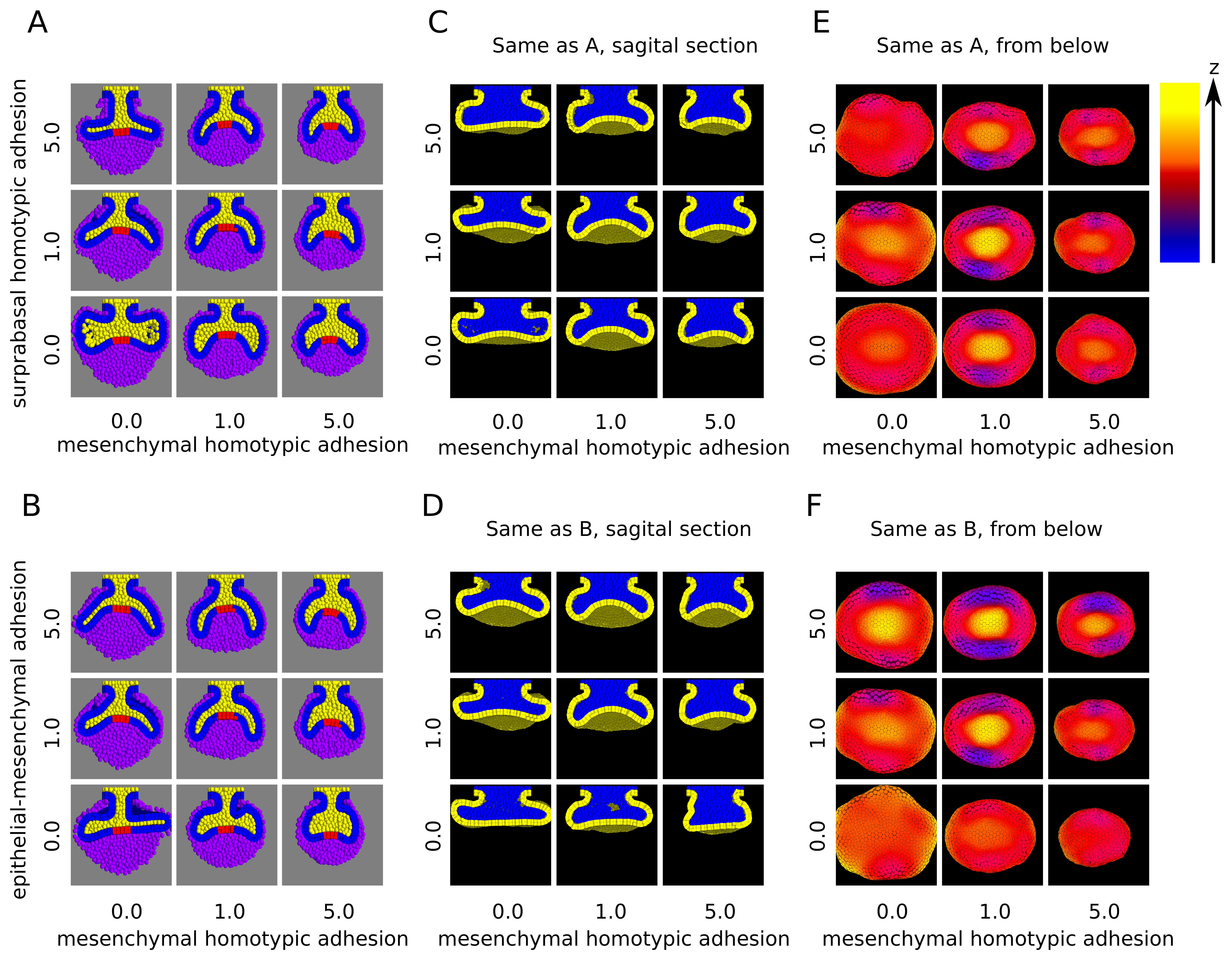

Supplement: S6 Fig — A, Tooth germ morphologies are shown with different combinations of mesenchymal homotypic adhesion and suprabasal homotypic adhesion values, while the other adhesion parameters are set to 1.0. High values of mesenchymal homotypic adhesion result in low growth angles whereas high values of suprabasal homotypic adhesion result in high growth angles. B, Morphologies are shown with different combinations of mesenchymal homotypic adhesion and epithelial-mesenchymal adhesion values (other adhesion parameters set to 1.0). High values of epithelial-mesenchymal adhesion result in low growth angles. Frontal sections shown. Epithelium in blue, suprabasal layer in yellow, mesenchyme in purple and enamel knot in red. C, D, Sagital sections of the same morphologies depicted in A and B respectively. Variation in adhesion parameters has the same effect in the anterior and posterior loops compared to the buccal and lingual, albeit the former tend to be shorter. Epithelium in yellow, suprabasal layer in blue, mesenchyme not shown. E, F, Same morphologies depicted in A and B respectively, only showing the epithelium and seen from below. The colour code indicates the heigth, or position in the Z axis. All the simulations in this screening used the following growth parameters: sepi = 0.063, ssup = 0.021, smes = 0.182. (PNG) [file pcbi.1005981.s006.png]

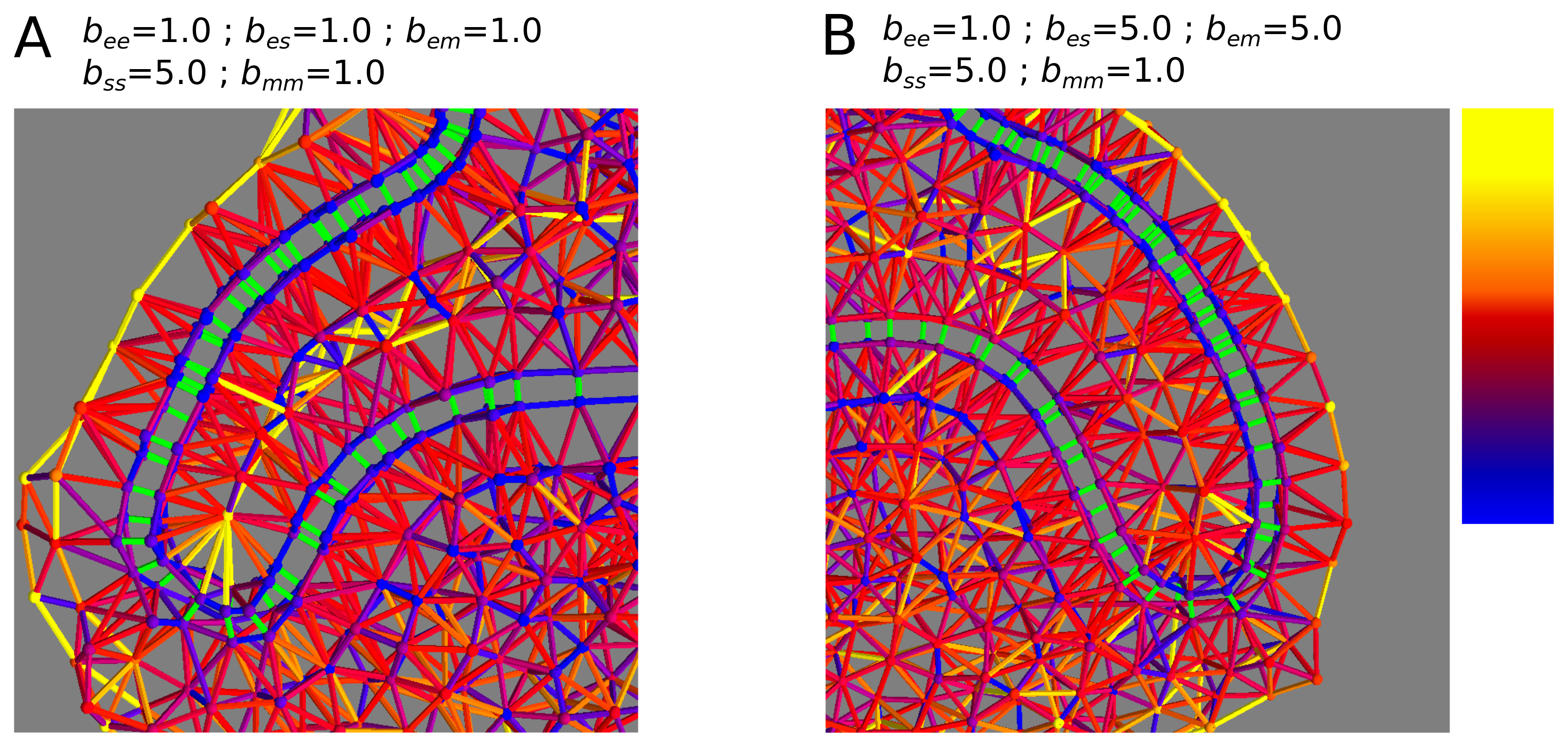

Supplement: S7 Fig — Epithelial cells are depicted as nodes (small spheres) connected by a spring (green). Thin frontal sections are shown in order to see clearly individual rods (i.e. force mechanical interactions. Parameters used in A, bee = 1.0, bes = 1.0, bem = 1.0, bss = 5.0, bmm = 1.0. Parameters used in B, bee = 1.0, bes = 5.0, bem = 5.0, bss = 5.0, bmm = 1.0. Colour coding is the same as in Fig 7. (PNG) [file pcbi.1005981.s007.png]

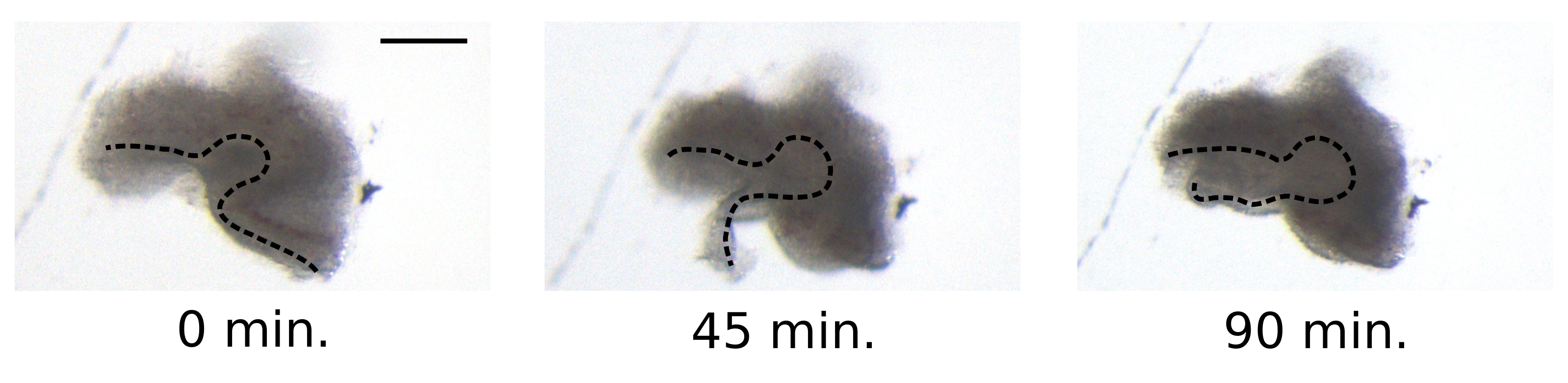

Supplement: S8 Fig — No major deformation in the epithelial bud is observed, and neither in the surrounding mesenchyme. Dashed line shows the epithelial mesenchymal boundary. Time after immersion in the dispase solution is shown beneath each panel. Scale bar is 200 μm long. (PNG) [file pcbi.1005981.s008.png]

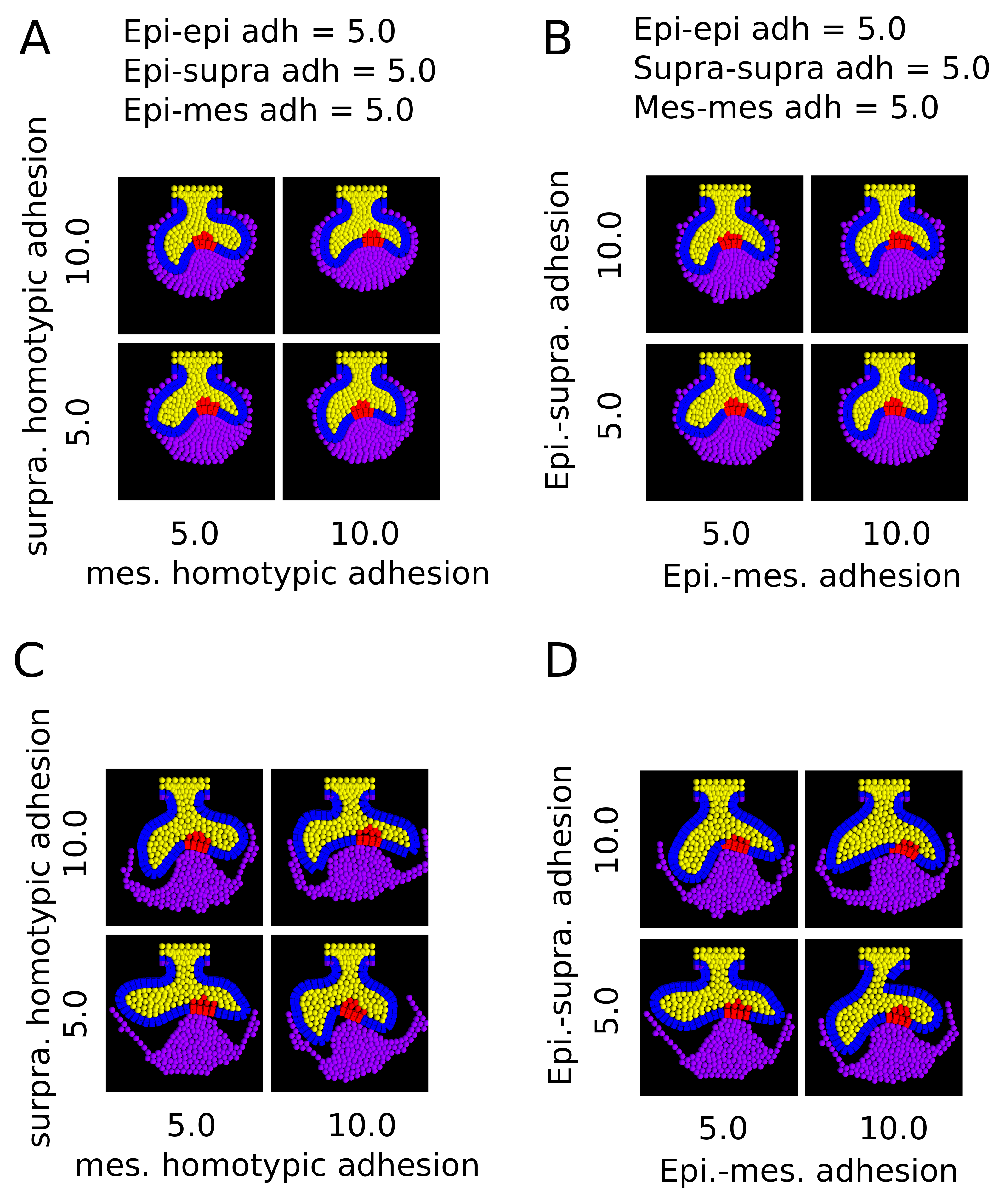

Supplement: S9 Fig — A, B, cap stage tooth germs simulated with different adhesion parameters. C, D, the same tooth germs as in A and B respectively, after the separation. Growth parameters used: sepi = 0.055, ssup = 0.033, smes = 0.245. (PNG) [file pcbi.1005981.s009.png]

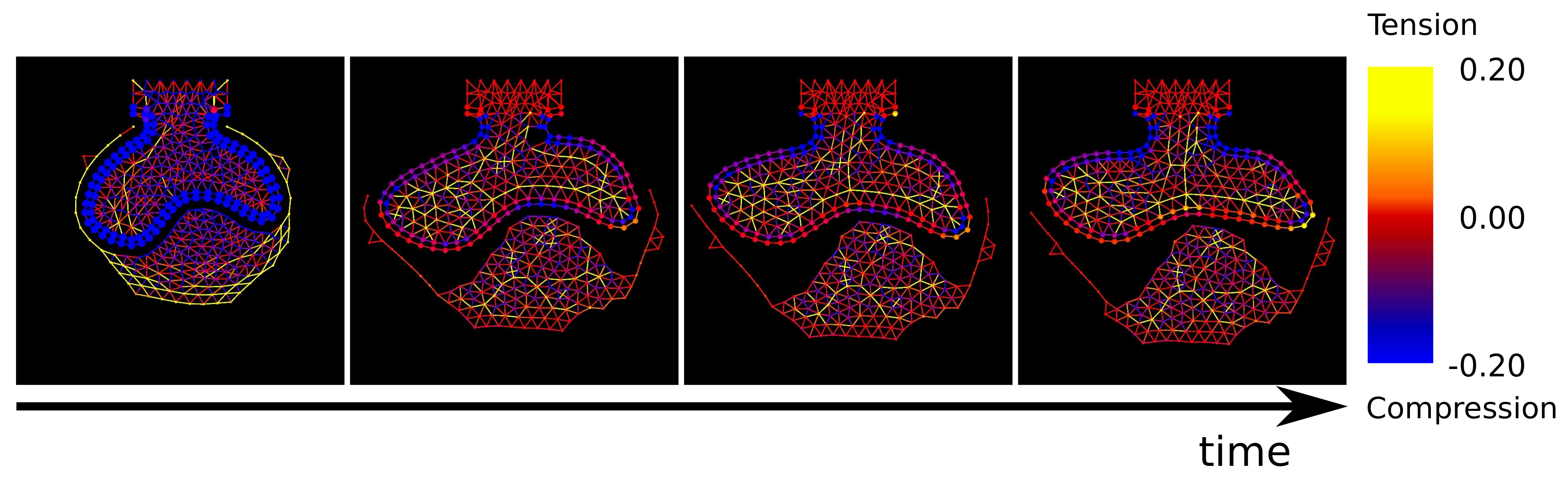

Supplement: S10 Fig — Colour code indicates mechanical stress, the same as in Figs 7 and 9. (PNG) [file pcbi.1005981.s010.png]

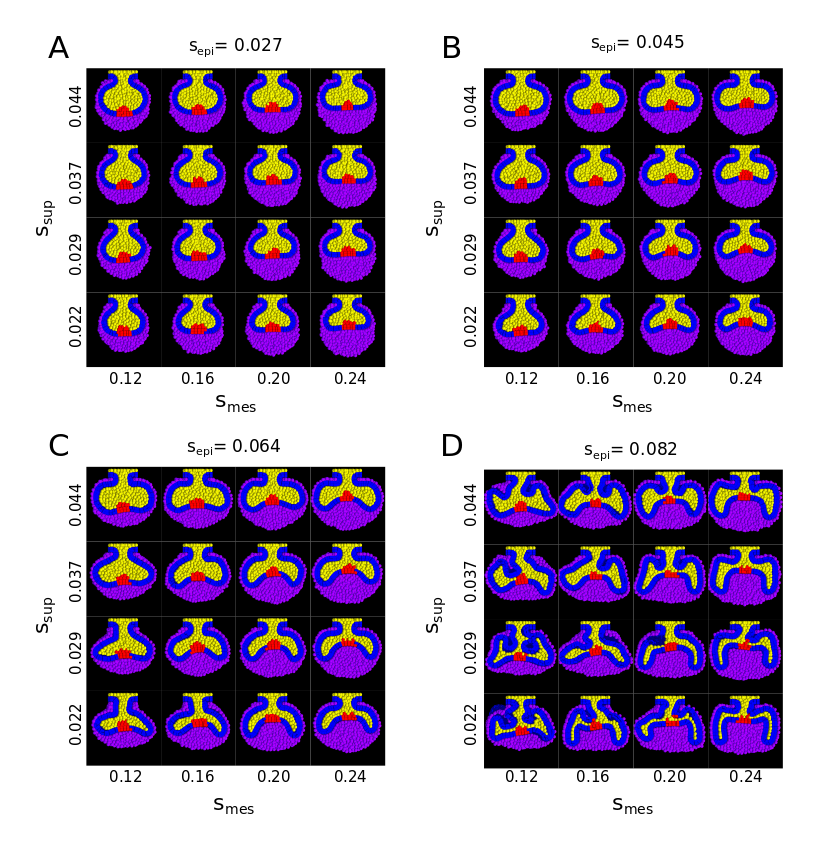

Supplement: S11 Fig — Each panel (A-D) displays model simulations for all the permutations of suprabasal and mesenchymal growth rate values (ssup and smes) for a constant value of epithelial growth rate (sepi). sepi values gradually increase in each subsequent panel. Colouring as in Fig 3. (PNG) [file pcbi.1005981.s011.png]

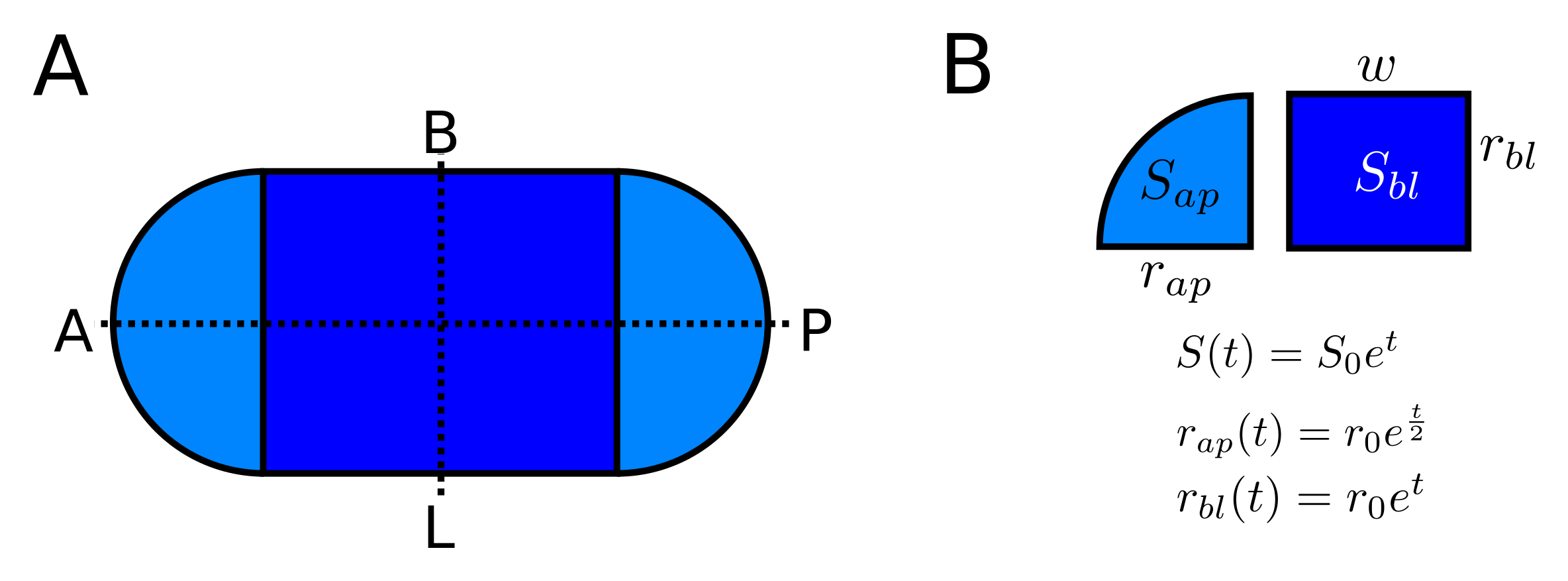

Supplement: S12 Fig — A, depiction of a simplified 2D tooth germ, seen from below. These could also be seen as tooth germs in which the angle of growth is 180 degrees. The light shade of blue indicates the anterior (A) and posterior (P) parts of the germ, where the A and P cervical loops are growing. The dark blue shade indicates the buccal (B) and lingual (L) portions of the tooth germ, where the B and L cervical loops are growing. B, the length of the cervical loops in the AP side (rAP) corresponds to the radius of a circle segment, whereas in the BL side (rBL) it corresponds to the side of a rectangle. Assuming that uniform tissue growth leads to an increase in surface area at a constant rate both in the AP and BL parts (SAP and SBL), the resulting elongation rates for the AP and BL cervical loops follow an exponential function with an exponent of t/2 and t respectively (t corresponds to time). (PNG) [file pcbi.1005981.s012.png]

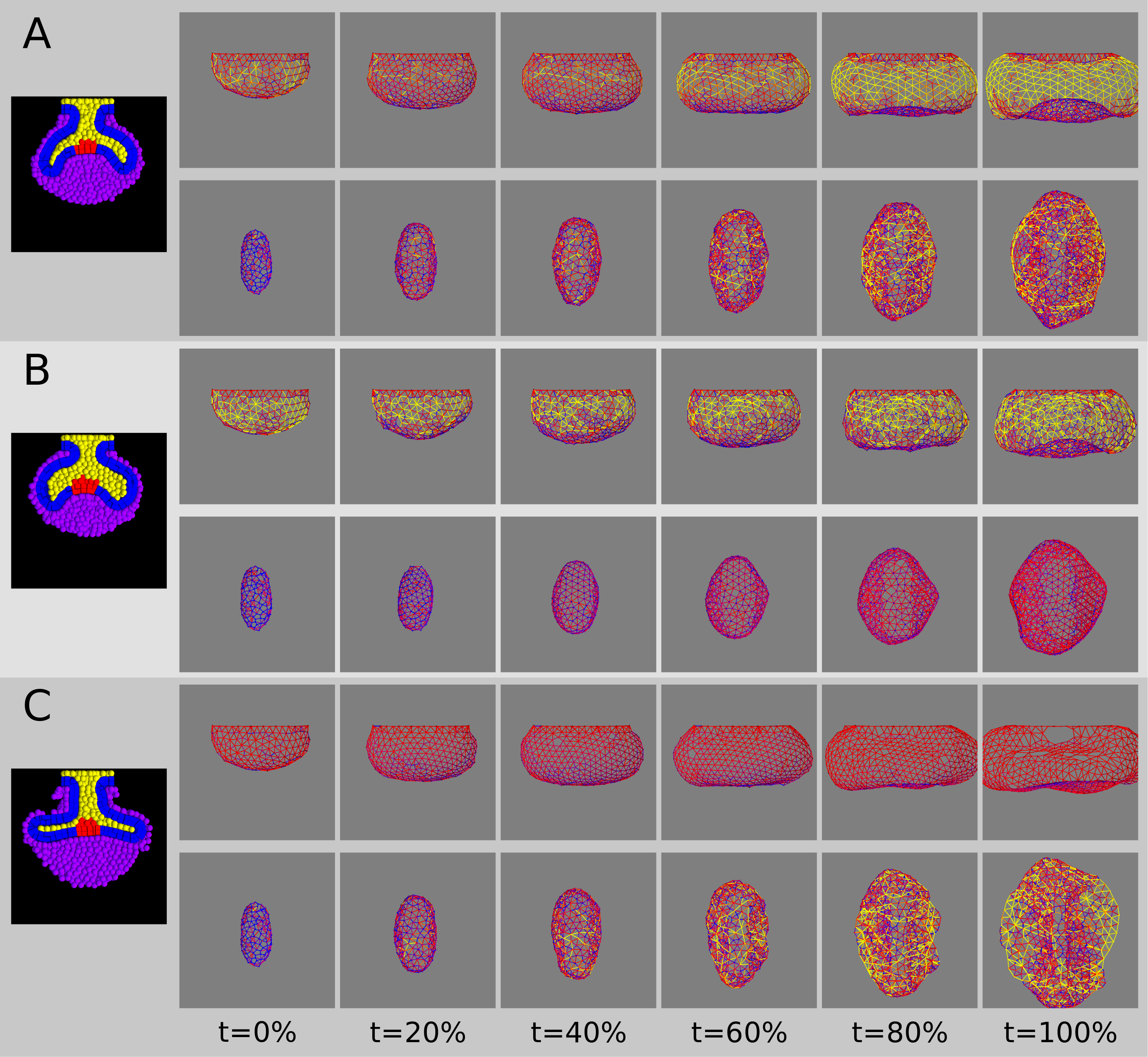

Supplement: S13 Fig — Time series of the three simulations (A-C) shown in Fig 7A–7C, depicting mechanical forces on the mesenchyme (top series for each panel) and the suprabasal layer (bottom series for each panel) at different time points. Top series for each simulation shows only the tooth mesenchyme through a sagittal cut (only the buccal half is displayed). Bottom series for each simulation shows only the suprabasal layer seen from below (as in Fig 7, right column). Rods follow the same colour coding as in Fig 7 (yellow for tension, blue for compression, red for null force). Left most pictures show the final morphology that corresponds to the ones displayed in Fig 7. (PNG) [file pcbi.1005981.s013.png]
