# Supplementary material for: Differential tissue growth and cell adhesion alone drive early tooth morphogenesis: An ex vivo and in silico study
Source: PLoS Comput Biol. 2018 Feb 26;14(2):e1005981. doi: 10.1371/journal.pcbi.1005981 (PMC5843354; doi:10.1371/journal.pcbi.1005981)
Supplement: S1 Table — SS, sum of squares; MS, mean square; Df, degrees of freedom; F, F-value; P, P value. (PDF) [file pcbi.1005981.s014.pdf]

Centroid size ANOVA					
**Effect**	**SS**	**MS**	**df**	**F**	**P**
Tooth germ	19895189.299353	41534.841961	479	2598.39	<0.0001
Digitising error	7672.715663	15.984824	480		
Shape, Procrustes ANOVA					
**Effect**	**SS**	**MS**	**df**	**F**	**P**
Tooth germ	52.16276651	0.0272248259	1916	139.1	<0.0001
Digitising error	0.3757972	0.0001957277	1920		
