# Supplementary material for: Differential tissue growth and cell adhesion alone drive early tooth morphogenesis: An ex vivo and in silico study
Source: PLoS Comput Biol. 2018 Feb 26;14(2):e1005981. doi: 10.1371/journal.pcbi.1005981 (PMC5843354; doi:10.1371/journal.pcbi.1005981)
Supplement: S1 Appendix — (PDF) [file pcbi.1005981.s016.pdf]

S1 Appendix

Mathematical implementation of EmbryoMaker features used in this model (adapted from Marin-Riera et al. 2016).

Nodes are containers of gene products. Gene products are produced by cells. Gene product transcription within a node is calculated as follows,

$$Q_{ik} = \frac{\Phi\left(\sum_{l=1}^{n_g} t_{lk} g_{il}\right)}{1+\Phi\left(\sum_{l=1}^{n_g} t_{lk} g_{il}\right)} \quad (8)$$

where Q_{ik} is the rate of transcription of gene k in node i, g_{il} is the amount of transcriptional factor l in node i and each t_{lk} term is the strength by which each specific transcriptional factor k activates (positive t_{lk}) or inhibits (negative t_{lk}) the transcription of gene l. Φ is a function that is equal to 0 for values of x smaller than 0 and equals to x when x is greater than 0 (Φ(x)=0 if x<0 and Φ(x)=x if x>0). This function is used to ensure that there is not such a thing as negative transcription (although t_{lk} can be negative and thus repress transcription). (see Marin-Riera et al. 2016 for a detailed explanation). Extracellular signal diffusion follows Fick's second law and takes place between nodes (see Marin-Riera et al. 2016 for a detailed explanation). Because of that, we ensure that there are never empty spaces within the tooth in the model system. Extracellular signals can interact with their corresponding receptor to activate signal transduction. In order to model the kinetics of ligand receptor binding we consider three different molecular species, the free ligand, the inactive receptor and the receptor-ligand complex. The kinetics of receptor-ligand binding are as follows,

$$S_{ic} = a_1 g_{il} g_{ik} - a_{-1} g_{ic} \quad (9)$$

where l is the free ligand, k is the free receptor, c is the receptor-ligand complex, S_{ic} is the rate of production of c on node i, g_{ix} is the concentration of gene product x on node i, and a_1 and a_{-1} are the forward and backward constants for the ligand-receptor binding reaction respectively and are set as model parameters. (see Marin-Riera et al. 2016 for a detailed explanation).

Diffusion is implemented as transfers of molecules between nodes,

$$O_{ik} = D_k \sum_{j=1}^{n_v} \left(\frac{g_{ik} - g_{jk}}{d_{ij}}\right) \quad (10)$$

Where g_{ik} is the amount of molecule k in node i, t is time, D_k is the diffusivity coefficient of molecule k, n_v is the number of neighbouring nodes.

Progression of the cell cycle depends is calculated as follows,

$$\frac{\partial P_h^{PHA}}{\partial t} = \frac{1}{n_h} \sum_{i=1}^{n_h} \sum_{m=1}^{n_g} c_m g_{im} \quad (11)$$

where c_m specifies the intensity with which gene product m regulates the cell cycle and g_{im} is the concentration of gene product m in node i (belonging to cell h). n_h is the number of nodes composing cell h (1 for mesenchymal and suprabasal cells and 2 for epithelial cells). In the tooth development model, each cell type had one specific gene that regulated cell proliferation (see next

section). Since expression levels of these genes (g_{im}) were usually constant over time, the rate of progression of cell cycle was mostly determined by the parameter c_m, and it is the parameter we changed when we wanted to change the growth rate of a certain tissue type. However, in some cases we wanted to suppress cell proliferation on cells that were expressing a division promoting gene, such as in the case of the signalling centre cells. In that case, a gene product specific to those cells was set with a large negative value of c_m, that suppressed the effect of the proliferative gene product as shown in equation 11.

Detailed description of the model initial conditions and parameters

All cells in the same tissue type express a specific gene product that acts as a transcription factor (TF), which also serves as a tissue specific marker. Each tissue specific TF promotes its own expression (see equation 8 in this section) ensuring that its expression is consistent over time and at a constant level. In turn, the tissue specific TF induces the expression of a tissue-specific adhesion molecule and a tissue-specific receptor for growth factors also at constant levels. A small group of epithelial cells located in the bucco-lingual mid line of the tooth bud is specified as a signalling centre, reminiscent of the primary enamel knot, by expressing a specific transcription factor.

In hypothesis I, each tissue specific TF regulates the progression of the cell cycle (and in hypothesis III this is the case only for epithelium and suprabasal layer). The intensity of that regulation by each TF can be specified independently through a model parameter, thus allowing to modulate tissue specific growth rates differentially. In hypotheses II and III, the signalling centre transcription factor promotes the production and secretion of an extracellular signal (from now on the growth factor) that diffuses to the surrounding cells and can interact with each tissue-specific receptor (epithelium, suprabasal and mesenchyme in B, and only mesenchyme in C). The binding between the growth factor an each type of receptor promotes the transcription of tissue specific effector genes that regulate the progression of the cell cycle in each tissue. The intensity with which each effector gene regulates the progression of the cell cycle is specified by a model parameter and by changing this in the different effector genes we can modulate the growth rate of each tissue independently.

Even though the signalling centre cells also express the epithelial specific TF, the signalling centre TF strongly represses the expression of the growth factor receptor, as has been shown experimentally and also represses the progression of the cell cycle directly.

In hypotheses II and III we create a distance dependent threshold for the activation of the tissue specific effector molecule so the effector is activated at roughly the same level in all cells within a certain distance of the signalling centre. We make that all tissue specific TF weakly repress the expression of the tissue specific effector molecule. This way, the effector will only be activated where a sufficient amount of growth factor reaches the cell and activates a sufficient amount of tissue specific receptors, so only the cells close enough to the signalling centre will activate the effector molecule.

Boundary conditions. The boundaries of the system were defined by; the edges of the epithelial sheet at the buccal, lingual, anterior and posterior sides of the tooth germ, the uppermost layer of suprabasal cells, and the outermost layers of mesenchymal cells. Spatial restrictions were imposed on some of these boundaries. Epithelial cells on the edges of the tissue were fixed in space, mimicking the mechanical resistance the surrounding oral epithelium would oppose. The suprabasal cells right on top of the epithelial boundary and the mesenchymal cells right under it are also fixed in space, representing the mechanical resistance presented by the surrounding tissues. A horizontal planar barrier was set on the uppermost boundary of the system that cells were unable to cross. The barrier was placed one cell diameter away from the apical side of the boundary epithelial cells, so there was enough space for one layer of suprabasal cells between the barrier and the boundary epithelial cells. The boundaries in the mesenchymal tissue were not fixed, so the cells were able to move downwards and towards the sides, except for the ones right under the epithelial boundary, that

were fixed. Overall, these boundary conditions prevented tooth germ growth towards the oral epithelium and upwards to the oral cavity, while allowing growth downwards and towards the buccal, lingual, anterior and posterior sides.

Default model parameters and node properties. The cell neighbouring method used was based on a Gabriel tessellation (Marin-Riera et al. 2016).

Epithelial cell plasticity (i.e. permanent deformation mediated by mechanical forces, see Marin-Riera et al. 2016) was deactivated in all simulations, thus all cells behaved like ideal elastic bodies.

Time progression was solved in all simulations by the Euler method, using a fixed time step size. All simulations have been run for 20000 time steps with a step size of 0,01 arbitrary time units. Since the real time-span between the bud and cap stages in the mouse molar is aproximately 48h, the arbitrary time unit in the model is equivalent to 14.4 min.

Node properties (see Materials and Methods and Marin-Riera et al. 2016) for all cell types were set to the following values unless indicated otherwise.

Node equilibrium radius: p^{EQD} = 0,25
Node interaction radius: p^{ADD} = 0,50
Node incompressibility coefficient p^{REC} = 0,50
Epithelial radial bending force coefficinet p^{EST} = 1,00
Epithelial rotational bending force coefficient p^{ERP} = 1,00
Epithelial spring equilibrium length p^{EQS} = 0,125
Epithelial spring elastic coefficient p^{HOO} = 5,00

Gene network and characterization of cell types.

A total of 31 gene products were specified in the system, with several gene-gene gene-cell behaviour interactions. Most of the gene products included in the model are devised to keep a constant expression over specific spatial territories (e.g. epithelium and mesenchyme), in order to differentiate them from one another and modify cellular properties differentially (e.g. growth). This gene network has been devised to implement the hypotheses and assumptions of our model (See main text) and, thus, we do not intend them to represent any known gene product expressed during tooth development.

The number of gene products as well as the gene-gene interactions in the model gene network are kept constant across all simulations and only the regulation of cell behaviours by these genes is varied. For example, in the implementation of hypothesis I, where all cells within a tissue divided at the same rate, that rate was regulated by a gene product expresses ubiquitously within a tissue (see below). In order to switch from Hypothesis I to II, in which cell proliferation was induced by signalling of a diffusible molecule produced by the enamel knot, we removed activation of proliferation from the ubiquitously expressed genes and made that specific genes that were activated in each tissue by the reception of the signal (see below) regulated cell division instead.

Degradation rate was set to 0.5 for all gene products and diffusion coefficient was set to 0.5 for all diffusive signals.

Varying the diffusion coefficient for the growth factor had the effect of varying the number of proliferative cells in the different tissues, thus it had the same effect as varying the rate of proliferation of cells which received the growth factor.

Varying the diffusion coefficient of the signalling centre expanding signal had the effect of varying the size of the signalling centre, which in turn meant varying the quantity and reach of the growth factor being secreted. Thus in only influenced the relative proliferation rates between cell types.

Parameter searches on p^{REC}, p^{EST} and p^{ERC}

When higher values of p^{REC} were set on a specific tissue, cells would tend to resist better the compression generated by surrounding tissue growth and thus occupy a larger volume with respect to cells with lower p^{REC} (Figure S2A-C). Since the effect of p^{REC} on overall tissue volume is saturating and rather equivalent to the effect of tissue-specifc growth rates, we decided to keep p^{REC} values constant and analyse the effect of growth rates in more depth. Epithelial bending parameters showed little effect on tooth germ shape unless they were set to 0, in which case the epithelium would break and the coherence of the tooth germ would be lost (Figure S2D). A slight reduction in cervical loop curvature was observed for high values of p^{ERP} and low values of p^{EST}, which can be explained by the effect of this parameters on the stiffness of the epithelial sheet (see Methods, also Marin-Riera et al. 2015).

Design of the 2D model of tooth development

The 2D version of the model was designed with exactly the same principles as the 3D version described in the previous section, with the difference that cells were initially distributed in a 2D plane reproducing the shape of a frontal section of the tooth bud (Figure 4A,B), and were allowed to move only within that plane (Figure 4C,D). The boundary conditions were the same as in the 3D model, the only difference is that in this case the epithelial boundary was only in the buccal and lingual sides of the tooth germ. In order to ensure the 2D model behaves similarly and thus is comparable to the 3D model, we checked whether the 2D and 3D models are able to produce similar tooth cap morphologies when using the exact same growth parameters (data not shown).

Estimation of growth curves of experimental time lapse from Morita et al. 2016 and model fitting.

A live-imaging time-lapse sequence of tooth development was published as supplementary material on the online version of Morita et al. 2016 (open access). We downloaded it as a movie file and split all the frames into separate image files.

We selected a time window (between frame 71 and 361, spanning 57h, Figure S4A, B) that actually encloses the stages we were modelling. We took one frame of every ten (separated from each other by roughly 6h) and measured the length of the epithelial layer and the surface area of the suprabasal layer by using the “segmented line” and “polygon selection” tools in Fiji (Schindelin et al. 2012). The outline of the interface between epithelium and suprabasal layer was drawn by hand (Figure S4C). From the resulting polygon, the surface area of the suprabasal layer was calculated. In order to calculate the length of the epithelium, the perimeter of the polygon was taken and the length of the uppermost segment (that does not include epithelium) was substracted (Figure S4C red segment). In order to be able to compare these measurements with the same ones done in the model, we chose as unit of length the maximum width of the tooth bud at the first time point that we measured (Figure S4A, white segment). By taking these two measures at each chosen time point we constructed two growth curves, one for the length of the epithelium and one for the surface area of the suprabasal layer.

We then devised a way to calculate the same growth curves in the 2D model of tooth development. For a single simulation, we selected specific time points that corresponded to the ones taken for the empirical growth curves (that is, they have the same relative position respect to the beginning and end of the time window) calculated a triangulated mesh for all the cells in the suprabasal layer (Figure S4E, mesh enclosed by cyan outline). The surface area of the suprabasal layer was calculated as the sum of the surface area of all the triangles in the mesh and the length of the epithelium as the sum of all the external edges of the mesh. Note that, as in the empirical case we also substracted the uppermost segments of the polygon that did not include epithelium (Figure

S4E, red segment). We took the maximum width of the tooth bud at the initial conditions of the model as unit of length so that the measurements are comparable with the empirical ones (Figure S4D, white segment).

In order to find a combination of model parameters whose growth curve best fit the empirical data, we used the 2D version of the model to generate a large parameter screening (n=9408) by permuting 6 different values (from lower to higher) for each of the 3 growth parameters and 2 different values (low and high) for each of the 5 adhesion parameters. We performed the same screening for the model version of hypothesis II and III. We obtained one epithelial and one suprabasal growth curve for each simulation. Then we proceeded to calculate the standard error (i.e. sum of squares) between each model growth curve and the corresponding empirical ones. We did that, for each growth curve, by calculating the square of the difference between each pair of corresponding points in the model and the empirical curve and finally summing the squared differences from all the time points. After getting the least squares scores for epithelium and suprabasal for all the simulations we sorted them from lowest to highest by looking at the sum of the two scores.

2D model cell movement tracking.

We tracked cell movements in the 2D model simulations by recording the position of each cell every 100 time steps (equivalent to 0.275 hours). In order to compare our in silico cell movements with Morita's experimental data we needed to focus on a time window in the model that was equivalent to the one chosen by Morita and collaborators in their experiments. The total time span considered in our model is roughly 60 hours, comprised between embryoninc day 13 and 15.5. The experimental data we used for this study consisted on a tooth germ cultured for 57 hours, corresponding roughly to the stages within embryonic days 13 and 15.5. The cell tracks shown corresponded to the last 20 hour period within the total 57 hour time span (that is roughly one third of the total time span). Thus, by plotting only the cell tracks corresponding to the last one third of the simulation duration we were able to generate in silico data that was comparable with Morita's experimental data.

Simplified model of antero-posterior vs. bucco-lingual growth of cervical loops.

In order to explain why in our model antero-posterior (AP) cervical loops do not grow as long as the bucco-lingual (BC) ones, we devised a simplified mathematical model. Let's assume that the cervical loops grow flat within the plane containting the AP and the BC axes (Figure S12A). For simplicity we will only consider one quarter of the tooth germ, delimited by the antero-posterior and bucco-lingual mid lines (Figure S12A). This quadrant can be split into two different shapes, one quarter of circle, corresponding to the antero-posterior border, and one rectangle corresponding to the tissue closer to the antero-posterior mid line. Growth of the former will contribute to the elongation of the antero-posterior cervical loops and growth on the latter will contribute to the elongation of the bucco-lingual cervical loops.

We assume that tissue growth is homogenous and is proportional to a constant division rate of the cells within. This means the rate of surface increase is proportional to the surface,

$$\frac{dS}{dt}=S \quad (12)$$

leading to an exponential function of growth.

$$S(t)=S_0 e^t \quad (13)$$

We also assume that the geometry of the tissue will be kept constant during growth, since this is what we observe in the tooth-specific model. By this we mean that, even though the morphology of the tooth germs changes substantially during growth, the curvature at the edges of the tooth epithelium is kept more or less the same (i.e. the antero-posterior cervical loops are more curved than the bucco-lingual ones). Thus, even though the same growth function holds true in both

portions of the tissue (the quarter of circle and the rectangle) in the simplified model, the rate of elongation of the tooth germ edge will follow different functions.

In the anterior-posterior portions of the tooth germ, the length of the tooth germ is equal to the radius of the quarter of circle, r_{ap} (Figure S12B). In the bucco-lingual portions, that length is determined by the width of the rectangle r_{bc} (Figure S12B). Thus, the relation between r_{ap} and r_{bc} and their respective surface areas is the following,

$$S_{ap}=\frac{\pi}{4}r_{ap}^{2} \quad (14)$$

$$S_{bl}=w\,r_{bl} \quad (15)$$

where w is the length of the rectangle in the antero-posterior direction. If we replace the surface area in the growth function with each of the equations above,

$$\pi r_{ap}^{2}=\pi r_{0ap}^{2}e^{t} \quad (16)$$

$$w\,r_{bl}=w\,r_{0bl}e^{t} \quad (17)$$

we get two different tooth edge elongation functions for the AP and the BC sides respectively when solved for r_{ap} and r_{bl} respectively,

$$r_{ap}=r_{0ap}e^{\frac{t}{2}} \quad (18)$$

$$r_{bl}=r_{0bl}e^{t} \quad (19)$$

where r_{0ap} *and* r_{0bl} are the length of the tooth germ edge at the start of growth for the AP and BL sides (t=0). Thus, the elongation of the AP edge of the tooth germ edge will be faster on the BC sides than on the AP sides simply due to the different geometries of these sides.

References

Marin-Riera M, Brun-Usan M, Zimm R, Välikangas T, Salazar-Ciudad I (2016) Computational modeling of development by epithelia, mesenchyme and their interactions: a unified model. *Bioinformatics* 32(2):219–25.

Morita R, et al. (2016) Coordination of Cellular Dynamics Contributes to Tooth Epithelium Deformations. *PLoS One* 11(9):e0161336.

Schindelin, J.; Arganda-Carreras, I. & Frise, E. et al. (2012), "Fiji: an open-source platform for biological-image analysis", Nature methods 9(7): 676-682
